# Glucagon-like peptide-1 receptor agonists, but not dipeptidyl peptidase-4 inhibitors, reduce alcohol intake

**DOI:** 10.1172/JCI188314

**Published:** 2025-03-06

**Authors:** Mehdi Farokhnia, John Tazare, Claire L. Pince, Nicolaus Bruns, Joshua C. Gray, Vincent Lo Re, David A. Fiellin, Henry R. Kranzler, George F. Koob, Amy C. Justice, Leandro F. Vendruscolo, Christopher T. Rentsch, Lorenzo Leggio

**Affiliations:** 1 Clinical Psychoneuroendocrinology and Neuropsychopharmacology Section, Translational Addiction Medicine Branch, National Institute on Drug Abuse Intramural Research Program and National Institute on Alcohol Abuse and Alcoholism Division of Intramural Clinical and Biological Research, NIH, Baltimore and Bethesda, Maryland, USA.; 2 Department of Mental Health, Johns Hopkins Bloomberg School of Public Health, Johns Hopkins University, Baltimore, Maryland, USA.; 3 Faculty of Epidemiology and Population Health, London School of Hygiene & Tropical Medicine, London, United Kingdom.; 4 Neurobiology of Addiction Section, Integrative Neuroscience Research Branch, National Institute on Drug Abuse Intramural Research Program, NIH, Baltimore, Maryland, USA.; 5 Stress & Addiction Neuroscience Unit, Integrative Neuroscience Research Branch, National Institute on Drug Abuse Intramural Research Program and National Institute on Alcohol Abuse and Alcoholism Division of Intramural Clinical and Biological Research, NIH, Baltimore, Maryland, USA.; 6 Department of Medical and Clinical Psychology, Uniformed Services University, Bethesda, Maryland, USA.; 7 Division of Infectious Diseases, Department of Medicine and Center for Clinical Epidemiology and Biostatistics, Perelman School of Medicine, University of Pennsylvania, Philadelphia, Pennsylvania, USA.; 8 Program in Addiction Medicine and; 9 Department of Internal Medicine, Yale School of Medicine, New Haven, Connecticut, USA.; 10 Department of Psychiatry, Perelman School of Medicine, University of Pennsylvania, Philadelphia, Pennsylvania, USA.; 11 Mental Illness Research, Education, and Clinical Center, Crescenz VA Medical Center, Philadelphia, Pennsylvania, USA.; 12 VA Connecticut Healthcare System, Department of Veterans Affairs, West Haven, Connecticut, USA.; 13 Center for Alcohol and Addiction Studies, Department of Behavioral and Social Sciences, Brown University, Providence, Rhode Island, USA.; 14 Division of Addiction Medicine, Department of Medicine, School of Medicine, Johns Hopkins University, Baltimore, Maryland, USA.; 15 Department of Neuroscience, Georgetown University Medical Center, Washington, DC, USA.

**Keywords:** Endocrinology, Neuroscience, Addiction, Peptides, Pharmacology

## Abstract

**BACKGROUND:**

Despite growing preclinical evidence that glucagon-like peptide1 receptor agonists (GLP-1RAs) could be repurposed to treat alcohol use disorder (AUD), clinical evidence is scarce. Additionally, the potential impact of dipeptidyl peptidase-4 inhibitors (DPP-4Is) on alcohol intake is largely unknown.

**METHODS:**

We conducted a large cohort study using 2008–2023 electronic health records data from the U.S. Department of Veterans Affairs. Changes in Alcohol Use Disorders Identification Test-Consumption (AUDIT-C) scores were compared between propensity-score–matched GLP-1RA recipients, DPP-4I recipients, and unexposed comparators. We further tested the effects of 2 DPP-4Is, linagliptin and omarigliptin, on binge-like alcohol drinking in mice and operant oral alcohol self administration in alcohol-dependent rats, models previously used to show a significant effect of the GLP-1RA semaglutide in reducing alcohol intake.

**RESULTS:**

GLP-1RA recipients reported a greater reduction in AUDIT-C scores than unexposed individuals (difference-in-difference [DiD]: 0.09 [95% CI: 0.03, 0.14], *P* = 0.0025) and DPP-4I recipients (DiD: 0.11 [95% CI: 0.05,0.17], *P* = 0.0002). Reductions in drinking were more pronounced among individuals with baseline AUD (GLP-1RA versus unexposed: 0.51 [95% CI: 0.29,0.72], *P* < 0.0001; GLP-1RA versus DPP-4I: 0.65 [95% CI: 0.43,0.88], *P* < 0.0001) and baseline hazardous drinking (GLP-1RA versus unexposed: 1.38 [95% CI: 1.07,1.69], *P* < 0.0001; GLP-1RA versus DPP-4I: 1.00 [95% CI: 0.68,1.33], *P* < 0.0001). There were no differences between DPP-4I recipients and unexposed individuals. The latter results were confirmed via a reverse translational approach. Specifically, neither linagliptin nor omarigliptin reduced alcohol drinking in mice or rats. The rodent experiments also confirmed target engagemhent, as both DPP-4Is reduced blood glucose levels.

**CONCLUSION:**

Convergent findings across humans, mice, and rats indicated that GLP-1RAs, but not DPP-4Is, reduce alcohol consumption and may be efficacious in treating AUD.

**FUNDING:**

This work was supported by the National Institutes of Health Intramural Research Program (ZIA DA000635, ZIA DA000644, ZIA DA000602), National Institute on Alcohol Abuse and Alcoholism extramural funding (R01 AA030041, P01 AA029545, U01 AA026224, U24 AA020794, U01 AA020790, U10 AA013566), the U.S. Department of Veterans Affairs (I01BX004820), and an Alkermes Pathways Research Award.

## Abstract 2

Consistent with preclinical data, real-world evidence indicates that receipt of GLP-1 receptor agonists, but not DPP-4 inhibitors, is associated with reduction in alcohol intake.

## Introduction

Alcohol use disorder (AUD) is associated with high morbidity and mortality. AUD treatments include psychosocial and pharmacological interventions (1, 2). The U.S. Food and Drug Administration (FDA) and the European Medicines Agency (EMA) have approved naltrexone, acamprosate, disulfiram, and nalmefene (the latter in Europe only) for treatment of AUD. Although these medications are efficacious, they are limited in number and not all patients respond to them (3, 4). Thus, expanding the armamentarium of pharmacotherapies for AUD is critical (5–8).

Increasing evidence, mostly from preclinical experiments and some preliminary human studies, suggests that glucagon-like peptide1 receptor agonists (GLP-1RAs), which are approved for treating type 2 diabetes mellitus and obesity, may be repurposed for AUD. This notion stems from a large body of basic neuroscience evidence on the role of the GLP-1 system in biobehavioral mechanisms that underlie alcohol misuse and addiction, including reward processing, stress regulation, and cognition (9–19), in addition to GLP-1’s well-known functions as an incretin and satiety hormone (20). GLP-1 is a 30 amino-acid peptide produced primarily in intestinal enteroendocrine cells and in the nucleus tractus solitarius (NTS) neurons. GLP-1 has a short half life of approximately 2 minutes and is rapidly degraded by the proteolytic enzyme dipeptidyl peptidase-4 (DPP-4). GLP-1 activates the GLP-1 receptor (GLP-1R), a G-protein coupled receptor expressed both in peripheral tissues, e.g., intestines, stomach, pancreas, liver, heart, and kidneys, and in the central nervous system, e.g., NTS, hypothalamus, nucleus accumbens, ventral tegmental area, amygdala, and hippocampus (21–28). Genetic variants that influence GLP-1R function have been associated with severity of alcohol use, risk of AUD, and brain functional activity/connectivity (29, 30). In a postmortem brain study, individuals with a history of AUD showed greater GLP-1R expression than controls in the hippocampus and prefrontal cortex (31).

Central or peripheral administration of GLP-1RAs reduces alcohol intake and other alcohol-related outcomes in mice, rats, and nonhuman primates (32–34). Compared with first-generation GLP-1RAs (e.g., exenatide), newer agents (e.g., semaglutide) are more potent and have longer half lives and higher receptor affinity (35, 36). Following our earlier preliminary study in male rats where liraglutide and semaglutide reduced alcohol intake and semaglutide also reduced alcohol preference (37), we recently showed that semaglutide dose dependently reduced binge-like alcohol drinking in mice and operant alcohol self administration in both alcohol-dependent and nondependent rats, with no sex differences (38). An independent study also found that semaglutide reduced alcohol intake and prevented relapse-like drinking in rats, using intermittent access and alcohol deprivation paradigms (39).

In the first clinical trial with a GLP-1RA in patients with AUD, exenatide (2 mg/week for 26 weeks), compared with placebo, had no significant effect on alcohol drinking outcomes in the full sample. In exploratory analyses from this study, exenatide reduced heavy drinking days and total alcohol intake in participants with a BMI over 30 kg/m^2^, whereas in those with a BMI under 25 kg/m^2^, an opposite effect was found. Of note, this trial had a high dropout rate (54.3%) (40). In a predefined secondary analysis of a smoking cessation clinical trial where all participants received varenicline and behavioral counselling and more than 90% had a BMI over 29.9 kg/m^2^, dulaglutide (1.5 mg/week for 12 weeks), compared with placebo, significantly reduced weekly alcohol consumption (41). Case series (42) and analyses of social media posts (43, 44) suggest that patients receiving semaglutide for diabetes or obesity may experience substantial reduction in alcohol use. However, these observations are preliminary and results from well-controlled human studies are needed (45).

Another relevant question is whether the putative effects of stimulating the GLP-1 system on alcohol intake may extend to medications that boost circulating endogenous GLP-1 levels via DPP-4 inhibition. Compared with GLP-1RAs, the effects of DPP-4 inhibitors (DPP-4Is), also approved for treating type 2 diabetes mellitus, on alcohol-related outcomes have been much less explored. In male rats, the DPP-4I sitagliptin delayed tolerance to anxiolytic-like effects of alcohol and withdrawal-induced anxiety-like behavior (46) but did not reduce alcohol intake or preference (37). Extreme reduction of DPP-4 activity, as measured by comparing DPP-4 deficient to wild-type F344 rats, was associated with less sensitivity to the sedative effects of alcohol but did not influence alcohol self administration (47).

In the present study, we investigated associations between the receipt of GLP-1RAs or DPP-4Is and changes in alcohol use in humans, using real-world electronic health record (EHR) data from the largest integrated healthcare system in the U.S., the Department of Veterans Affairs (VA). We applied propensity-score matching between exposure and comparator groups and conducted difference-in-difference (DiD) analyses on Alcohol Use Disorders Identification Test-Consumption (AUDIT-C) scores. AUDIT-C is a self-reported 3-item questionnaire on alcohol use frequency and quantity; it is validated and widely used as a screening tool with scores denoting severity of alcohol use (48, 49). To further corroborate our human findings, we also examined the effects of 2 DPP-4Is, one that does not cross the blood-brain barrier (linagliptin) and one that does (omarigliptin) (50–53), on alcohol intake in mice and rats, using the same models we previously used to show a significant effect of the GLP-1RA semaglutide in reducing alcohol intake (38).

## Results

### Effects of GLP-1RAs and DPP-4Is on alcohol consumption in humans

#### Sample.

**Figure 1:**
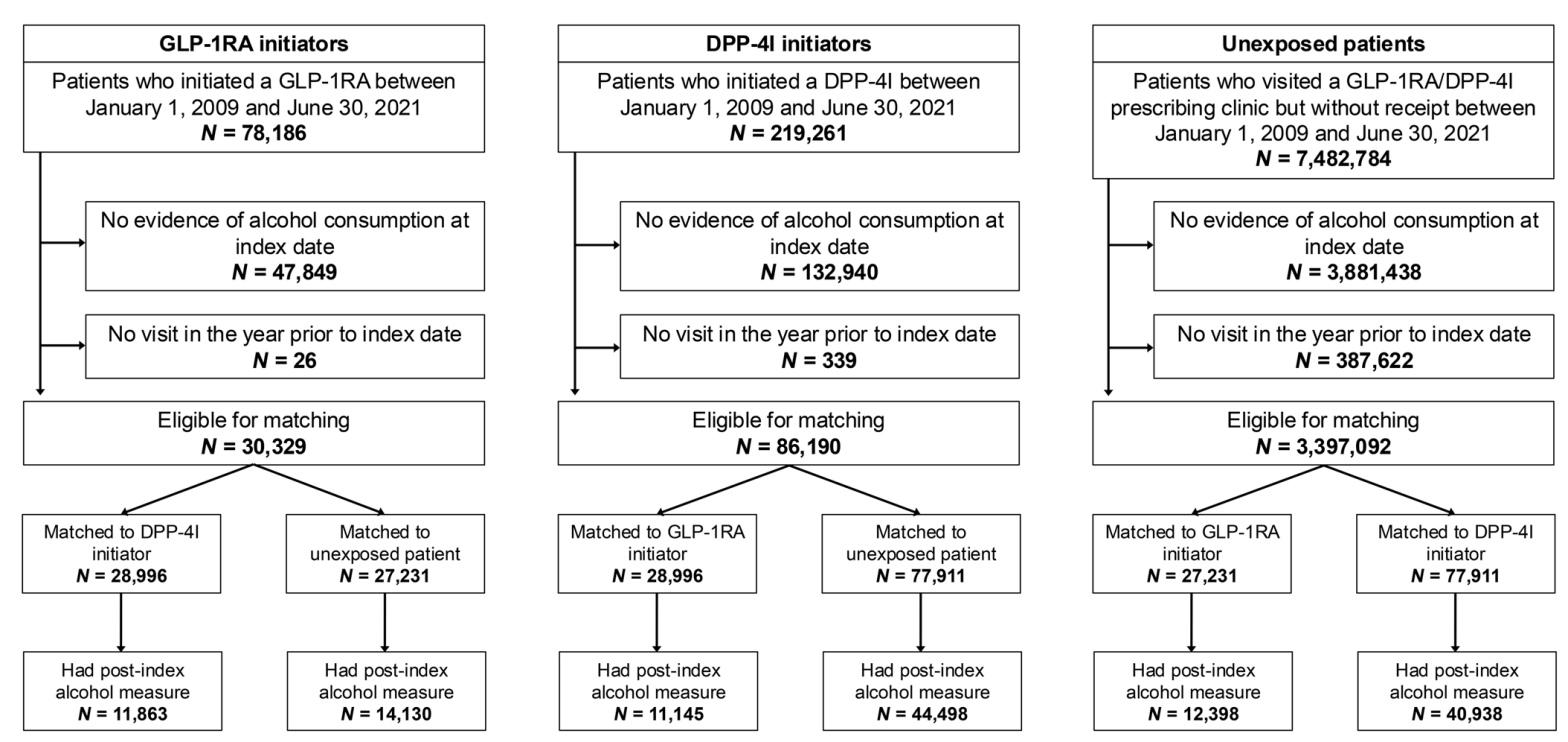
Flow diagram of the human cohort study. Numbers presented for excluded individuals are not mutually exclusive.

Figure 1 presents a flow diagram of the study. We identified 30,329 GLP-1RA recipients, 86,190 DPP-4I recipients, and 3,397,092 eligible unexposed comparators who reported any alcohol consumption in the 2 years prior to the index date. Propensity-score matching was performed separately for each exposure contrast and resulted in 27,231 individuals per group for GLP-1RA versus unexposed (referred to as “contrast A”), 77,911 for DPP-4I versus unexposed (referred to as “contrast B”), and 28,996 for GLP-1RA versus DPP-4I (referred to as “contrast C”). After excluding those without an eligible follow-up AUDIT-C, the final analytic cohorts included 14,130 GLP-1RA recipients and 12,398 unexposed for contrast A (Supplemental Table 1; supplemental material available online with this article; https://doi.org/10.1172/JCI188314DS1), 44,498 DPP-4I recipients and 40,938 unexposed for contrast B (Supplemental Table 2), and 11,863 GLP-1RA recipients and 11,145 DPP-4I recipients for contrast C (Supplemental Table 3).

Before propensity-score matching, the distribution of baseline characteristics differed between groups. After propensity-score matching and restricting participants to those with a post-index AUDIT-C score, groups were well balanced (standardized mean differences [SMDs] ≤ 0.1; Supplemental Tables 1–3). Distribution of propensity scores for each exposure contrast before and after matching are depicted in Supplemental Figure 1.

#### Changes in alcohol consumption — Contrast A.

**Figure 2:**
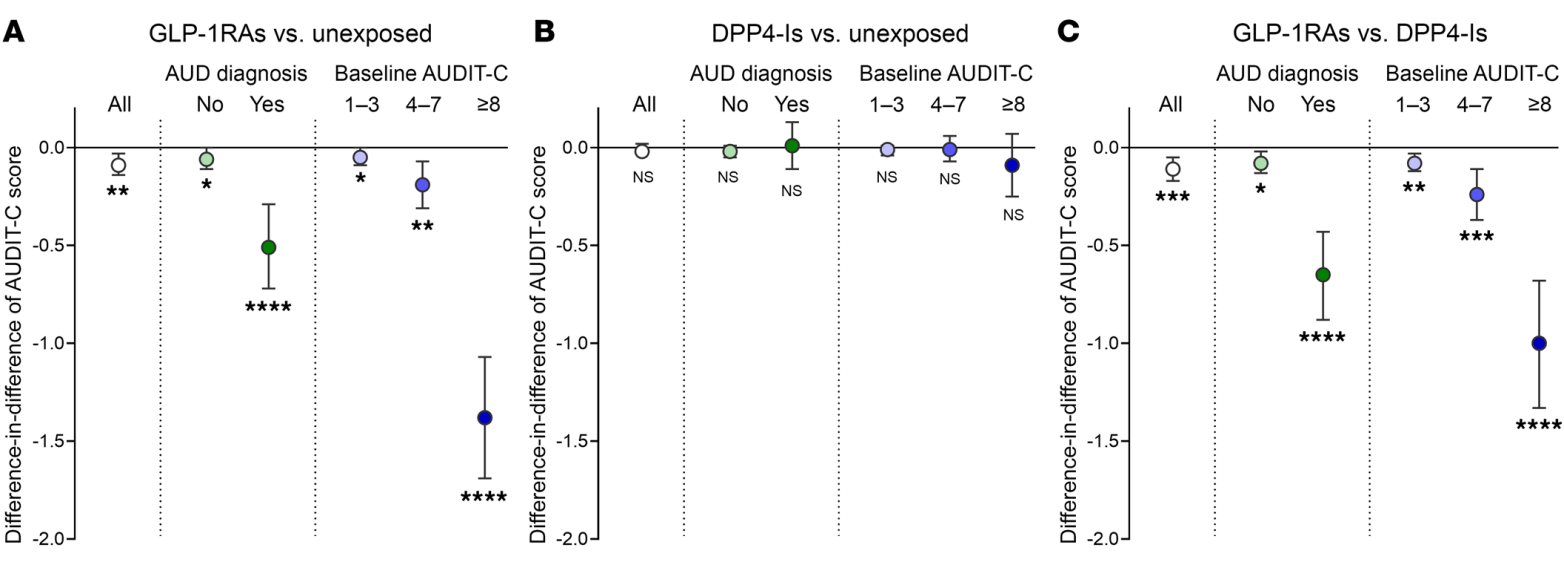
Association between receipt of GLP-1RAs or DPP-4Is and alcohol use in humans. Difference-in-difference estimates and 95% confidence intervals of changes in AUDIT-C scores, overall (white) and stratified by baseline AUD diagnosis (green) and by baseline AUDIT-C score (blue). (**A**) GLP-1RA recipients versus unexposed individuals, (**B**) DPP-4I recipients versus unexposed individuals, (**C**) GLP-1RA recipients versus DPP-4I recipients. *P < 0.05, **P < 0.01, ***P < 0.001, ****P < 0.0001.

Contrast A is shown in Figure 2A. GLP-1RA recipients showed a significantly greater reduction in average AUDIT-C scores than unexposed comparators (DiD: 0.09 points, 95% CI: 0.03,0.14; *P* = 0.0025) (Table 1). This effect was more pronounced among individuals with baseline AUD (DiD: 0.51 points, 95% CI: 0.29,0.72; *P* < 0.0001) (Supplemental Table 4) and those with baseline hazardous drinking, i.e., baseline AUDIT-C ≥ 8 (DiD: 1.38 points, 95% CI: 1.07,1.69; *P* < 0.0001) (Supplemental Table 5).

#### Changes in alcohol consumption — Contrast B.

Contrast B is shown in Figure 2B. No significant differences in AUDIT-C score changes were found between DPP-4I recipients and unexposed comparators, either overall or in subgroup analyses (Table 1 and Supplemental Tables 4–6), indicating no effect of DPP-4Is on alcohol consumption.

#### Changes in alcohol consumption — Contrast C.

Contrast C is shown in Figure 2C. GLP-1RA recipients showed a significantly greater reduction in average AUDIT-C scores than DPP-4I recipients (DiD: 0.11 points, 95% CI: 0.05,0.17; *P* = 0.0002) (Table 1). This effect was more pronounced among individuals with baseline AUD (DiD: 0.65 points, 95% CI: 0.43,0.88; *P* < 0.0001) (Supplemental Table 4) and those with baseline hazardous drinking (DiD: 1.00 point, 95% CI: 0.68,1.33; *P* < 0.0001) (Supplemental Table 5).

Analyses stratified by baseline BMI showed no differential patterns in AUDIT-C DiD based on BMI (Supplemental Table 6). Results were similar to analyzing GLP-1RAs as a class when GLP-1RA exposure was restricted to semaglutide, though with wider confidence intervals (Supplemental Table 7 and Supplemental Figure 2).

### Effects of DPP-4Is on alcohol consumption in rodents

#### Linagliptin or omarigliptin had no effect on binge-like alcohol drinking while lowering blood glucose levels in mice.

**Figure 3:**
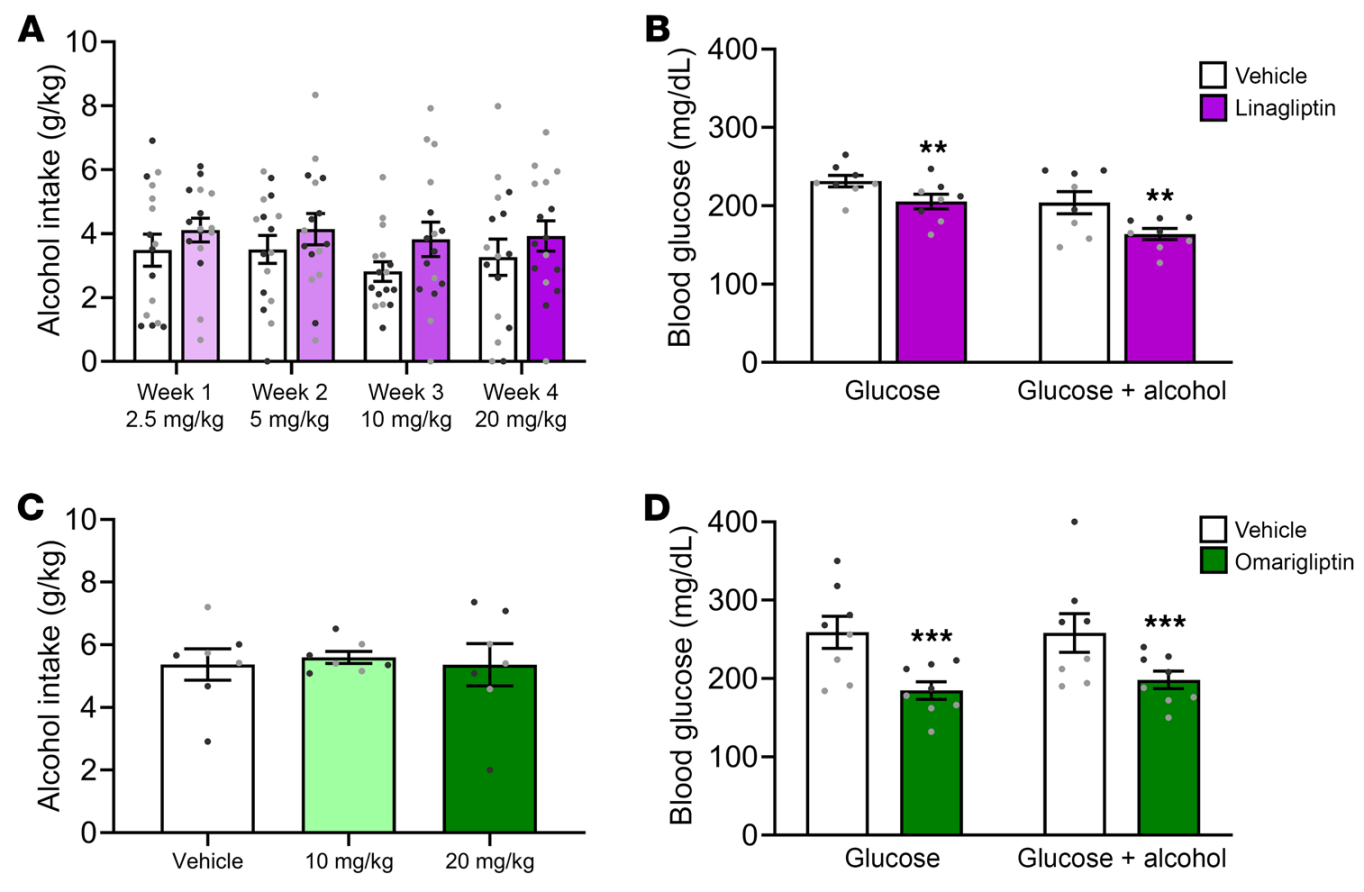
Effects of DPP-4Is on alcohol intake and blood glucose levels in mice. (**A**) Linagliptin (2.5, 5, 10, and 20 mg/kg, s.c.), tested using a between-subjects design (see Figure 5A), had no effect on binge-like alcohol drinking, measured on the first 4-hour session (Tuesday) of each week, in mice (*n* = 16 males, 16 females). Drug (linagliptin) effect: F_1,28_ = 1.90, *P* = 0.18; week effect: F_3,8_ = 1.04, *P* = 0.38; sex effect: F_1,28_ = 0.80, *P* = 0.38, drug × week interaction: F_3,84_=0.16, *P* = 0.93; drug × sex interaction: F_1,28_=0.05, *P* = 0.82; week × sex interaction: F_3,84_ = 2.12, *P* = 0.10; drug × week × sex interaction: F_3,84_ = 1.07, *P* = 0.37. (**B**) Linagliptin (20 mg/kg, i.p.), tested using a between-subjects design, lowered blood glucose levels following both glucose (*n* = 8 males, 8 females) and glucose-plus-alcohol (*n* = 8 males, 8 females) challenge tests in mice. Drug (linagliptin) effect: F_1,28_ = 11.16, *P* = 0.002; alcohol effect: F_1,28_ = 12.19, *P* = 0.002 (glucose alone > glucose plus alcohol); drug × alcohol interaction: F_1,28_ = 0.52, *P* = 0.48. (**C**) Omarigliptin (10, 20 mg/kg, i.p.), tested using a within-subjects design (see Figure 5B), had no effect on binge-like alcohol drinking in mice (*n* = 4 males, 3 females). Drug (omarigliptin) effect: F_2,10_ = 0.04, *P* = 0.96; sex effect: F_1,__5_= 0.51, *P* = 0.51; drug × sex interaction: F_2,10_ = 0.52, *P* = 0.61. (**D**) Omarigliptin (20 mg/kg, i.p.), tested using a between-subjects design, lowered blood glucose levels following both glucose (*n* = 8 males, 8 females) and glucose plus alcohol (*n* = 8 males, 8 females) challenge tests in mice. Drug (omarigliptin) effect: F_1,28_ = 13.99, *P* = 0.0008; alcohol effect: F_1,28_ = 0.12, *P* = 0.73; drug × alcohol interaction: F_1,28_ = 0.16, *P* = 0.69. Individual data symbols are shown in black for males and in gray for females. Data are expressed as mean (SEM). **P < 0.01, ***P < 0.001.

No drug, week, sex, or interaction effects were found on binge-like alcohol drinking, measured on the first 4 hour session (Tuesday) of each week, in mice treated weekly with dose-escalating s.c. linagliptin (Figure 3A). Linagliptin did not change alcohol intake on Fridays either (Supplemental Figure 3). Data were analyzed separately to differentiate between potential acute (day of injection on Tuesdays) and delayed (no injection on Fridays) linagliptin effects. Linagliptin (s.c.) did not affect body weight throughout this 4-week experiment (Supplemental Figure 4). We also tested i.p. linagliptin and found no effect on binge-like alcohol drinking in mice (Supplemental Figure 5). Similarly, omarigliptin (i.p.) did not change binge-like alcohol drinking in mice (Figure 3C). Confirming target engagement and related pharmacological effects, both s.c. linagliptin (Figure 3B) and i.p. omarigliptin (Figure 3D) lowered blood glucose levels in mice following i.p. administration of glucose alone or glucose plus alcohol.

#### Linagliptin or omarigliptin had no effect on operant alcohol self administration in alcohol-dependent rats.

**Figure 4:**
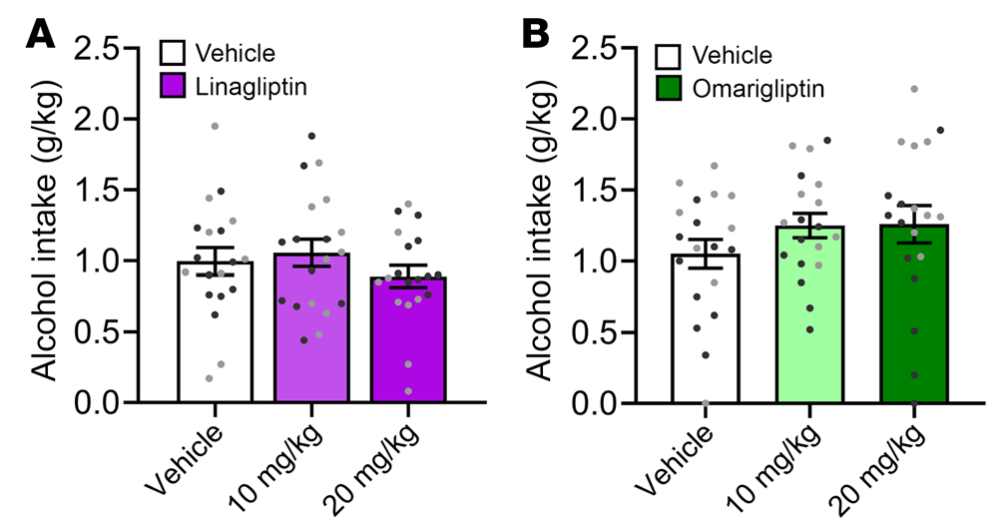
Effects of DPP-4Is on alcohol intake in rats. (**A**) Linagliptin (10, 20 mg/kg, i.p.), tested using a within-subjects design (see Figure 5C), had no effect on operant oral alcohol self administration in alcohol-dependent rats (*n* = 10 males, 9 females). Drug (linagliptin) effect: F_2,34_ = 2.01, *P* = 0.15; sex effect: F_1,17_ = 0.18, *P* = 0.68; drug × sex interaction: F_2,34_ = 1.75, *P* = 0.19. (**B**) Omarigliptin (10, 20 mg/kg, i.p.), tested using a within-subjects design (see Figure 5C), had no effect on operant oral alcohol self administration in alcohol-dependent rats (*n* = 10 males, 9 females). Drug (omarigliptin) effect: F_2,34_ = 1.73, *P* = 0.19; sex effect: F_1,17_ = 6.99, *P* = 0.02 (female > male); drug × sex interaction: F_2,34_ = 0.82, *P* = 0.45. Individual data symbols are shown in black for males and in gray for females. Data are expressed as mean (SEM).

No drug or drug × sex interaction effects were found on alcohol intake during operant oral alcohol self administration in alcohol-dependent rats treated with i.p. linagliptin (Figure 4A) or i.p. omarigliptin (Figure 4B). Similarly, the number of alcohol deliveries and water intake were unchanged by linagliptin or omarigliptin (Supplemental Figure 6).

## Discussion

The development, approval, and rapid clinical adoption of GLP-1 medications has revolutionized the management of diabetes and obesity. Following several years of basic neuroscience research, evidence on the promise of these medications for neuropsychiatric conditions such as addiction and neurodegenerative disorders is rapidly growing (54–56). Consistent data across different laboratories and animal models indicate that GLP-1RAs reduce alcohol intake and other alcohol-related outcomes in rodents and nonhuman primates (32–34). To move the field forward, we aimed to translate these findings to humans, using real-world evidence, and found that receipt of GLP-1RAs was associated with a significant reduction in alcohol use. The magnitude of this effect was most robust in people with AUD and those with hazardous alcohol drinking at baseline. Receipt of GLP-1RAs was associated with a significant reduction in AUDIT-C even among people without AUD and with lower levels of drinking. Consistent with our previous finding that semaglutide reduces alcohol intake in both alcohol-dependent and nondependent rodents (38), these results suggest that GLP-1RAs may help people across a broad spectrum of alcohol use, misuse, and use disorder. By contrast, our pharmacoepidemiologic findings do not support a beneficial role for DPP-4Is in reducing alcohol consumption. To confirm our negative human findings with DPP-4Is, we took a reverse translational approach and further tested linagliptin and omarigliptin in rodents, using the same models that previously showed a significant effect of semaglutide in reducing alcohol intake (38). Consistent with our human findings, DPP-4Is had no effect on alcohol intake in mice or rats. If anything, alcohol drinking was slightly higher under DPP-4Is versus vehicle in some of our rodent experiments (e.g., Figure 3A and Figure 4B), although no statistically significant differences were observed.

For our human cohort study, we used real-world EHR data from the VA and applied propensity-score matching to ensure balance on important covariates across the 3 exposure groups. As expected in a middle-aged cohort engaged in a healthcare setting, AUDIT-C scores decreased over time in all 3 groups. However, this reduction was significantly greater among GLP-1RA recipients than both DPP-4I recipients and unexposed comparators (i.e., those who did not receive either GLP-1RAs or DPP-4Is). By contrast, receipt of DPP-4Is was not associated with changes in AUDIT-C scores, a finding replicated in our rodent experiments. In a Danish nationwide register-based study, receipt of GLP-1RAs, compared with DPP-4Is, was associated with a lower incidence of alcohol-related events, including hospital contacts with a main diagnosis of AUD, registered treatments for AUD, and purchase of AUD or alcohol withdrawal pharmacotherapy (57). This Danish study, unlike ours, did not include a measure of alcohol use severity and did not have an unexposed comparator group. Another study, using built-in functions within the TriNetX Analytics Platform, found that receipt of semaglutide, compared with other non-GLP-1RA medications for obesity and/or diabetes, was associated with reduced incidence and recurrence of AUD (58). A more recent study applied a discovery approach in VA databases to compare people with diabetes who initiated GLP-1RAs and those on other non-GLP-1RA diabetes medications, and found that the use of GLP-1RAs was associated with reduced risk of alcohol and other substance use disorders, among several other health outcomes (59). The present pharmacoepidemiologic study complements and expands these previous findings by including 3 exposure groups (GLP-1RA recipients, DPP-4I recipients, and unexposed individuals), looking at both GLP-1RAs as a class and semaglutide individually, and using harmonized data from an integrated healthcare system that enhances reliability and reduces the risk of missing data and confounding factors such as receiving care outside the network. In addition, a unique aspect of the VA EHR data used here is the ability to analyze changes in AUDIT-C as an outcome (rather than just AUD diagnosis), given its roughly annual collection on all patients during routine healthcare visits. AUDIT-C is a well-established alcohol screening tool that provides a quantitative and continuous measure of alcohol use across the spectrum (48, 49).

Our analyses found considerable decreases in alcohol use from baseline to follow-up under GLP-1RAs, as indicated by robust DiD estimates. Among those who reported hazardous alcohol use (AUDIT-C ≥ 8) at baseline, GLP-1RAs as a class and semaglutide alone were associated with at least a 1-point reduction in AUDIT-C scores on average. These observed reductions are larger than prior pharmacoepidemiologic analyses in VA cohorts (60–62), suggesting a strong effect of GLP-1RAs on alcohol intake. Exposure groups were balanced on baseline and demographic characteristics, including baseline severity of alcohol use, as indicated by SMDs equal to or less than 0.1. For example, in the final cohort of the GLP-1RA versus DPP-4I comparison, SMDs of low risk (AUDIT-C of 1–3), at-risk (AUDIT-C of 4–7), and hazardous (AUDIT-C of ≥ 8) drinking at baseline were 0.003, 0.003, and less than 0.001, respectively (Supplemental Table 3), indicating proper balance and no difference across groups. It is important to note that most people in this sample were in the low-risk drinking category (overall baseline AUDIT-C score around 2, Table 1), and a more robust effect with GLP-1RAs could be expected in samples with higher severity of alcohol use. In our definition of exposure, we ensured that patients across groups had comparable follow-up periods. For example, in the GLP-1RA versus DPP-4I comparison, the median active exposure was 271 and 285 days for GLP-1RA and DPP-4I initiators, respectively (Supplemental Table 3). It was not feasible to conduct a detailed dose-response analysis, mainly because GLP-1RAs and DPP-4Is follow different dose scheduling and formulations (e.g., most of the GLP-1RAs are injectable, whereas DPP-4Is are oral). Even within the class of GLP-1RAs, different medications have different dose scheduling, require an uptitration period, and clinicians often change the dose, depending on tolerability, side effects, and clinical response. Therefore, imputing dose information and conducting dose-response analyses would be misleading in this case. The optimal safe and effective dose of GLP-1RAs that may reduce alcohol drinking remains to be determined.

It is also important to study predictors of response and identify subgroups of patients who may benefit the most from GLP-1RAs. BMI has been suggested as a potential moderator (40), but our pharmacoepidemiologic study showed no clear pattern when analyses were stratified by BMI. There have also been some preclinical reports of sex differences in drinking behavior in response to GLP-1RAs, but the results have been inconclusive. For example, a recent study with semaglutide showed a greater reduction in alcohol intake in female rats (39), whereas dulaglutide and exendin-4 appeared to be more effective in males (63, 64). We tested male and female mice and rats here and in our previous semaglutide study (38) and found no sex differences. We were unable to stratify our pharmacoepidemiologic analyses by sex since more than 90% of the VA cohort was male. Nevertheless, evaluating potential sex differences remains an important question, especially considering some sex-divergent effects of GLP-1 in the CNS (65, 66). It is also important to note that the VA cohort predominantly comprises older individuals (with the most frequent group being 60–69 year olds; Supplemental Tables 1–3), further limiting the sample size of premenopausal females.

**Figure 5:**
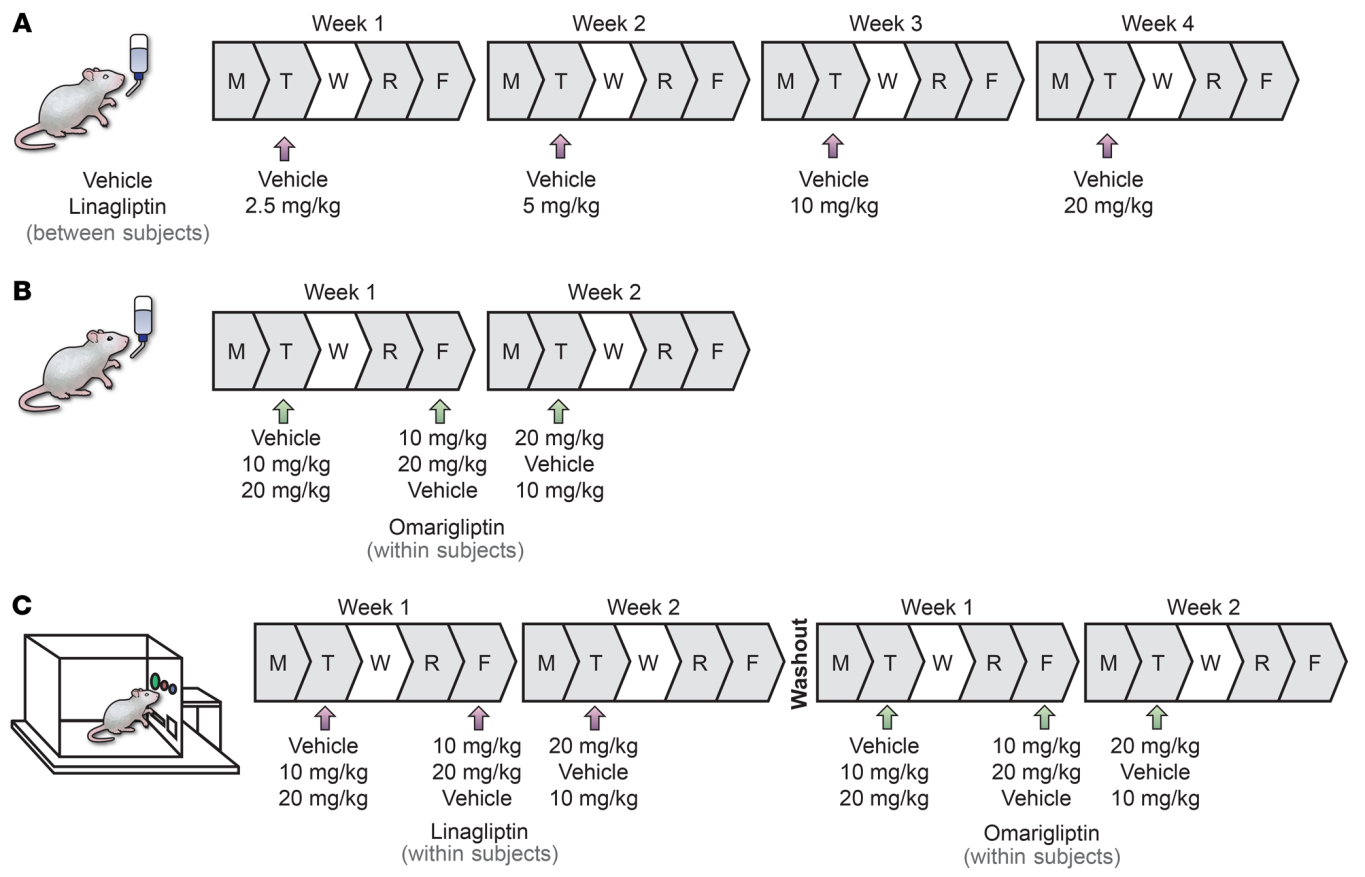
Schematics of the main rodent experiments. (**A**) Effect of linagliptin on drinking-in-the-dark in mice was tested using a between-subjects design. Mice were assigned to 1 of the 2 groups: vehicle or linagliptin. The vehicle group received vehicle once a week for 4 weeks, whereas the linagliptin group received escalating doses of linagliptin (2.5, 5, 10, and 20 mg/kg, s.c.), 1 injection per week (Tuesdays). Sweetened alcohol solution was given for 4 hours on Tuesdays (results in Figure 3A) and Fridays (results in Supplemental Figure 3) and for 2 hours on Mondays and Thursdays (data not shown). (**B**) Effect of omarigliptin on drinking-in-the-dark in mice was tested using a within-subjects design. Mice received vehicle and 2 doses of omarigliptin (10, 20 mg/kg, i.p.) in a randomized (Latin-square) order on each 4-hour drinking test day (Tuesday/Friday; results in Figure 3C). Sweetened alcohol solution was given for 2 hours on Mondays and Thursdays (data not shown). (**C**) Effects of linagliptin and omarigliptin on operant oral self administration in alcohol-dependent rats were tested using a within-subjects design. Rats were first made dependent using alcohol vapor exposure. They received daily, intermittent cycles of 14 hours of alcohol vapor exposure and 10 hours off (withdrawal). Operant oral alcohol self administration was performed 6–8 hours into withdrawal. Male rats were tested first with linagliptin then omarigliptin (as shown in the figure). Female rats were tested first with omarigliptin then linagliptin (opposite of the order shown in the figure). Linagliptin and omarigliptin testing was separated by at least 4 days (washout). Rats received vehicle and 2 doses of linagliptin (10, 20 mg/kg, i.p.; results in Figure 4A) or vehicle and 2 doses of omarigliptin (10, 20 mg/kg, i.p.; results in Figure 4B) in a randomized (Latin-square) order on each test day (Tuesday and Friday). Alcohol intake was measured after each 30-minute, fixed ratio 1, operant self-administration session.

The first rodent experiment we conducted was s.c. linagliptin testing in mice (drinking-in-the-dark model). Given the relatively long half-life of linagliptin (67, 68), we chose a between-subjects design for this experiment (Figure 5A). In such a design, if linagliptin changed drinking on a Tuesday testing (i.e., the first 4-h drinking session of the week, Figure 3A), we would be able to look for a “carry over” effect on Friday (i.e., the second 4-hour drinking session of the week with no linagliptin injection, Supplemental Figure 3). Because we did not find an effect on drinking on week 1 (2.5 mg/kg), we doubled the dose on week 2 (5 mg/kg) and repeated the same schedule on week 3 (10 mg/kg) and week 4 (20 mg/kg). Mice that received vehicle once a week served as controls. Given the lack of effect of linagliptin in this first experiment and to reduce the number of animals without lowering the study’s scientific validity and rigor, we subsequently used within-subjects designs to test different doses of omarigliptin in the same mice (Figure 5B) and different doses of linagliptin and omarigliptin in the same rats (Figure 5C), similar to our previous study with semaglutide (38).

The preclinical paradigms employed here model different aspects and stages of alcohol use. The drinking-in-the-dark model leads to binge-like alcohol intake and often produces intoxication (69). The alcohol vapor model simulates alcohol dependence and produces motivational and somatic signs of withdrawal (70). Along with preliminary evidence from prior work (37, 46, 47), the present negative results across 3 species provide strong evidence that DPP-4 inhibition is not an effective intervention for reducing alcohol intake. We conducted a control experiment showing that, as expected, both linagliptin and omarigliptin reduced blood glucose levels following a glucose challenge test in fasted mice. Based on some prior evidence in humans (31, 71), we asked whether alcohol may reduce circulating endogenous GLP-1 levels to a point that DPP-4Is do not have enough substrate to exert an effect. Thus, we repeated the glucose challenge with alcohol coadministration, and, again, linagliptin and omarigliptin significantly reduced blood glucose levels. Therefore, the lack of effect of DPP-4Is on alcohol drinking in our rodent experiments appears to be a ‘true negative’ finding that cannot be attributed to a potential lack of target engagement.

In addition to the gut, GLP-1 is produced in the CNS, and studies suggest that peripherally administered GLP-1 can cross the blood-brain barrier (BBB) (72, 73). The ability of GLP-1 medications to cross the BBB and be taken up in the brain has been a topic of research and some controversy. Most DPP-4Is, including linagliptin, cannot cross the BBB and are peripherally restricted (52). After finding no effect on alcohol drinking with linagliptin, we asked whether these negative results could be attributed to the drug’s inability to cross the BBB and to reach the brain. Thus, we tested omarigliptin, a DPP-4I that can cross the BBB (53). Consistent negative findings indicate that DPP-4Is do not impact alcohol drinking regardless of their brain penetrance. Relatedly, several studies have shown that small, nonacylated, non-PEGylated GLP-1RAs such as exenatide and dulaglutide can cross the BBB, while larger and more complex ones such as liraglutide and semaglutide may not be able to cross an intact BBB (74–77). While additional research is needed to characterize brain uptake pharmacokinetics of these GLP-1 medications, it is important to note that efficacy for CNS conditions like AUD does not necessarily require active transport across the BBB for at least 3 reasons. First, effects in the periphery and peripheral-central signals have been shown to mediate some beneficial effects of GLP-1 medications (78–80). Second, many chronic conditions such obesity and AUD lead to a disruption of the BBB and may increase its permeability to these medications (81–83). For example, Aranäs and colleagues detected fluorescently labelled semaglutide, after acute i.p. administration, in the nucleus accumbens of mice chronically exposed to alcohol. They hypothesized that alcohol-related changes in the BBB facilitated the ability of systemic semaglutide to reach the nucleus accumbens (39). Third, there are brain structures bordering the third and fourth ventricles, namely circumventricular organs (CVOs), with no functional BBB that are highly sensitive to neuroendocrine signals. For example, the median eminence in the hypothalamus and the area postrema in the hindbrain are 2 brain regions with the highest GLP-1R density (84). These CVOs are recruited by GLP-1 and GLP-1RAs and facilitate signaling to adjacent and more distal brain regions, including those involved in alcohol use and other addictive behaviors (76, 85, 86).

GLP-1RAs are generally analogs to the endogenous GLP-1 peptide but with longer half-lives, more potency, and higher receptor affinity (87, 88). Unlike GLP-1RAs, increasing endogenous GLP-1 levels by inhibiting DPP-4 does not seem sufficient to reduce alcohol intake. While GLP-1 release is stimulated by food intake, circulating GLP-1 levels are also detectable during fasting, suggesting a tonic level of activity (89–91). Low basal levels of GLP-1 may theoretically lead to insufficient levels for there to be an impact on alcohol intake, even in the presence of DPP-4Is. The drinking-in-the-dark model in mice included mild food restriction, with ad libitum access to chow until the beginning of the 4-hour test sessions, during which mice had access only to the sweetened alcohol solution. Neither linagliptin nor omarigliptin changed alcohol self administration in rats either, despite much shorter food restriction during drinking sessions (30 minutes). Thus, feeding status does not seem to play a role in DPP-4Is’ lack of effect on alcohol intake. DPP-4Is are approved for the treatment of type 2 diabetes mellitus; they provide glycemic control by increasing GLP-1 levels and insulin release and reducing glucagon secretion and hepatic glucose output. However, DPP-4Is have minimal or no effect on appetite, feeding/consummatory behavior, or body weight, which is why, unlike GLP-1RAs, DPP-4Is are not approved for treating obesity. In addition to enhancing glycemic control, GLP-1RAs inhibit gastric emptying and their anorectic properties are thought to be primarily driven by actions in the CNS, either directly (e.g., via GLP-1Rs in several brain regions) and/or through peripheral-central signals (e.g., via vagal afferent neurons) (78, 80, 89, 92–96). These mechanistic differences between GLP-1RAs and DPP-4Is likely contribute to the divergent effects of these drugs on alcohol-related outcomes. Several neurobiological mechanisms have been proposed to underly GLP-1RAs’ beneficial effects in reducing alcohol use and other addictive behaviors, including their impact on reward processing (9–12), stress regulation (13–15), appetitive and consummatory behaviors (97, 98), thirst and fluid intake (99–101), cognitive function and neuroprotection (16, 17, 56), pain (102), aversion (103, 104), and neuroinflammation (105–107), among others. Understanding whether/how these mechanisms contribute to the impact of GLP-1RAs on alcohol intake requires additional work.

Collectively, compelling evidence across multiple species indicate that GLP-1RAs, but not DPP-4Is, reduce alcohol intake and are promising candidates for treating AUD. Randomized controlled trials remain the gold standard of proof for drug efficacy and are critically needed at this juncture to investigate the safety and efficacy of GLP-1RAs in individuals with AUD and/or other substance use disorders (34, 45).

## Methods

### Sex as a biological variable

Both sexes were included in the human and rodent studies. Sex differences could not be examined in the pharmacoepidemiologic analyses due to the high male-to-female ratio in the VA cohort (> 90%, Supplemental Tables 1–3). Sex differences were examined in rodent experiments and no sex differences were observed.

### Real-world evidence in humans

#### Data source.

We extracted data from the Veterans Aging Cohort Study–National (VACS-National), which includes approximately 13.5 million Veterans who ever received care in the U.S. Department of VA. The VA is the largest integrated healthcare system in the U.S., serving approximately 9 million patients annually at more than 1,300 hospitals, medical centers, and outpatient clinics nationwide (108). All care is recorded in EHR with daily uploads into the VA corporate data warehouse. Available data include demographics, diagnoses (ICD-9/-10 codes), pharmacy dispensing records, laboratory results, procedures, vital signs, height, weight, and routinely collected measurements of smoking and alcohol consumption. This study is compliant with the Health Insurance Portability and Accountability Act and is reported following guidelines for strengthening the reporting of observational studies in epidemiology (STROBE) and reporting of studies conducted using observational routinely collected health data (RECORD) (Supplemental Appendix 1).

#### Exposure groups.

Our study included 3 groups: GLP-1RA recipients, DPP-4I recipients, and unexposed individuals. We identified new initiators of GLP-1RAs (exenatide, albiglutide, dulaglutide, liraglutide, and semaglutide) during the study period, requiring a 180-day washout period to ensure new exposure episodes. Exposure to GLP-1RAs was defined as receipt of 2 or more doses, for any indication, between January 1, 2009 and June 30, 2021. New initiators of DPP-4Is (alogliptin, saxagliptin, sitagliptin, and linagliptin) were identified using the same criteria. For constructing the unexposed group, we first identified outpatient clinics that were the largest sources of GLP-1RA and DPP-4I prescriptions. We then selected all individuals who attended at least one of these clinics, but never received a GLP-1RA or DPP-4I, to ensure that unexposed individuals came from the same source population, were exposed to similar medical care overall, and had an opportunity to receive a GLP-1RA or DPP-4I. We randomly selected one visit per unexposed individual to be carried forward in the analyses.

Index date was defined as the first dispensed date for GLP-1RA or DPP-4I recipients and the randomly selected outpatient visit date for unexposed individuals. We excluded individuals with no outpatient care in the year prior to index date due to the inability to capture baseline data, individuals with no measurement of alcohol consumption in the 2 years prior to index date, and those who reported no alcohol consumption based on the closest measurement to index date.

#### Covariates.

We extracted information on a wide range of potential confounders, including age at baseline, race, ethnicity, sex, urban/rural residence, geographic region, year of index date, and history of clinical conditions/procedures prior to baseline, including AUD, opioid use disorder, posttraumatic stress disorder, bariatric surgery, diabetes, cancer, asthma, chronic obstructive pulmonary disease, congestive heart failure, myocardial infarction, peripheral vascular disease, peptic ulcer, liver disease, renal disease, cerebrovascular disease, dementia, hemiplegia/paraplegia, rheumatic disease, human immunodeficiency virus (HIV) infection, Charlson Comorbidity Index, and VACS Index (version 2.0). The Charlson Comorbidity Index is a mainstay measure of overall comorbidity based on diagnostic codes across 17 clinical domains (109, 110). The VACS Index is a summary score that assesses physiologic frailty using a validated algorithm that primarily incorporates routinely available laboratory measures (111, 112). We also derived variables that captured exposure to other medications, including medications with demonstrated effects on alcohol consumption (naltrexone, acamprosate, disulfiram, gabapentin, topiramate, varenicline, and spironolactone) (7, 8, 61, 113), any neurocognitive-active or high-burden anticholinergic medications (114), and total number of chronic medications. Other potential confounders included substance use treatment program visits, smoking status, BMI, systolic and diastolic blood pressure, laboratory measures (albumin, total cholesterol, high-density lipoprotein [HDL] cholesterol, triglycerides, total bilirubin, hemoglobin, glycated hemoglobin [HbA1c], white blood cell count, fibrosis-4 [FIB-4] score, and estimated glomerular filtration rate [eGFR]). Lastly, we created variables denoting whether the index prescription/visit was in primary care, total number of visits to a prescribing clinic, total number of visits to any clinic, and any hospitalization in the 2 years prior to index date.

#### Propensity-score matching.

Propensity-score matching was performed to balance the distribution of potential confounders across groups. Propensity scores (i.e., predicted probability of exposure) were estimated using 3 multivariable logistic regression models, each modelling 1 of the 3 exposure contrasts of interest: GLP-1RA versus unexposed (referred to as “contrast A”, DPP-4I versus unexposed (referred to as “contrast B”), and GLP-1RA versus DPP-4I (referred to as “contrast C”). Of 54 variables in each model, most (38) had complete data, only 3 had 10%–15% missingness, and all others had 10% or less missingness. We included a missing category for covariates with missing data. Under the additional assumption that associations between fully observed covariates and exposure do not differ across missingness patterns, this approach produces unbiased estimates (115, 116). The *C*-statistic was 0.99, 0.98, and 0.76 for models A, B, and C, respectively, indicating adequate discrimination between groups. Within each exposure contrast, each individual from 1 group was matched to 1 individual from the other group on the logit of the propensity score with a caliper of 0.20 times the standard deviation (SD) ​​of the logit of the propensity score in the region of common support and using a greedy matching algorithm (117). Individuals were exactly matched on AUD diagnosis and baseline AUDIT-C categories.

#### Outcome and follow up.

Our primary outcome of interest was change in alcohol consumption using AUDIT-C (Supplemental Appendix 2). AUDIT-C includes 3 questions, each scored from 0–4. The resulting AUDIT-C scores range from 0–12, with the likelihood of alcohol-related morbidity and mortality increasing as scores increase (48, 49, 118–120). An AUDIT-C score of 0 indicates no current alcohol use, 1–3 suggests low-risk drinking, 4–7 suggests at-risk drinking, and ≥ 8 suggests hazardous or heavy episodic alcohol use. Since 2008, the VA has required annual AUDIT-C screening for all individuals in primary care (121).

Participants were followed for a maximum of 2 years from their index date or until their last VA visit, death, or June 30, 2023. Additionally, GLP-1RA and DPP-4I recipients were censored at their last received dose. To ensure equal follow-up time within matched pairs, unexposed comparators were censored at the total follow-up time of their matched exposed individual. Although evidence of alcohol consumption at baseline (i.e., AUDIT-C score > 0) was an inclusion criterion, availability of a follow-up AUDIT-C was not required for matching eligibility as such a restriction would not translate to an analogous prospective clinical trial.

#### Statistics.

Absolute SMDs were calculated to examine balance between each exposure contrast in the unmatched, matched, and final analytic cohort after restricting the matched cohorts to those with outcome measurement; SMDs equal to or less than 0.1 indicated balance (122). Among individuals in the final analytic cohort, we calculated the average pre- and post-index AUDIT-C scores. Pre-index AUDIT-C scores were defined as the closest on or before the index date, within a maximum of 2 years prior. Post-index AUDIT-C scores were defined as the measure during and closest to the end of follow-up. We then used multivariable DiD linear regression models (123, 124) to estimate the differential change between pre- and post-index AUDIT-C scores for each exposure contrast. We also performed subgroup analyses stratified by current AUD diagnosis (i.e., within 2 years prior to the index date), baseline AUDIT-C, and baseline BMI. Finally, we reran the analyses restricting GLP-1RA exposure to semaglutide as one of the most promising drugs in preclinical alcohol studies (38, 39) with several ongoing randomized controlled trials for AUD (e.g., NCT06015893, NCT05891587, NCT05520775, NCT05892432, and NCT05895643). Microsoft SQL Server Management Studio v18.11 and SAS Enterprise Guide v8.3 (SAS Institute) were used for data management and analysis, respectively.

### Rodent experiments

#### Animals.

Adult, male and female C57BL/6J mice were acquired from Jackson Laboratory and weighed 17–25 g at baseline. Adult male and female Wistar rats were acquired from Charles River Laboratories and weighed 420–600g at baseline. Mice were single housed; rats were group housed (2–4 per cage). Mice and rats were housed in standard cages and in temperature- and humidity-controlled rooms with a reverse 12 hour/12 hour light/dark cycle (lights off at 7:00 a.m.) and given ad libitum access to food and water except during behavioral testing, which occurred during the dark cycle. All procedures were performed in accordance with the National Institutes of Health Guide for the Care and Use of Laboratory Animals and approved by the Institutional Animal Care and Use Committee of the National Institute on Drug Abuse Intramural Research Program.

#### Drugs.

Linagliptin (Cayman Chemical) and omarigliptin (TargetMol) were prepared with 5% Tween 80 (v/v; Thermo Fisher Scientific) and 0.9% saline. The volume of injection was 10 mL/kg for mice and 1 mL/kg for rats.

Figure 5 illustrates the experimental timeline and design of the rodent studies. For binge-like alcohol drinking (drinking-in-the-dark test) in mice, linagliptin was initially administered s.c. in a between-subjects (linagliptin versus vehicle; *n* = 8 males, 8 females, per treatment group), dose-escalating (2.5, 5, 10, and 20 mg/kg) fashion. Mice in the vehicle group received vehicle weekly throughout the 4-week experiment, while mice in the linagliptin group received escalating doses of linagliptin weekly throughout the 4-week experiment (Figure 5A).

We also tested i.p. linagliptin on binge-like alcohol drinking in mice. For this experiment, mice that were tested with repeated s.c. linagliptin/vehicle over 4 weeks (mentioned above) were given a 2-week break from linagliptin/vehicle and alcohol drinking for washout. They were then reassigned to linagliptin or vehicle groups matched by sex and baseline drinking and were given a single i.p. injection of linagliptin (20 mg/kg) or vehicle, again in a between-subjects fashion and tested for binge-like alcohol drinking.

In a separate cohort of mice (*n* = 4 males, 3 females), we tested omarigliptin on binge-like alcohol drinking. For this experiment, we used a within-subjects design in which each mouse received vehicle and 2 doses of omarigliptin (10, 20 mg/kg, i.p.) in different testing days in a randomized (Latin-square) order (Figure 5B).

In separate cohorts of mice (*n* = 4 males, 4 females, per drug condition, per experiment), the highest doses of linagliptin (20 mg/kg, i.p.) and omarigliptin (20 mg/kg, i.p.) were tested in glucose-challenge tests in the absence and presence of alcohol to confirm target engagement and expected pharmacological effects to reduce peripheral glucose levels. These experiments used a between-subjects design, in which mice received either linagliptin or vehicle, or omarigliptin or vehicle.

We also tested linagliptin and omarigliptin in a different species and model, i.e., operant self administration in alcohol-dependent rats (*n* = 10 males, 9 females). For these experiments, we used a within-subjects design in which each rat received vehicle and 2 doses of linagliptin (10, 20 mg/kg, i.p.) or omarigliptin (10, 20 mg/kg, i.p.) in different testing days in a randomized (Latin-square) order (Figure 5C). Male rats were tested first with linagliptin then omarigliptin. Female rats were tested first with omarigliptin then linagliptin. At least 4 days of washout was given between linagliptin and omarigliptin testing. Doses were chosen based on previous literature (125–127) and our mouse experiments mentioned above.

#### Drinking-in-the-dark test in mice.

A drinking-in-the-dark test was used to model alcohol binge-like drinking in mice (61, 69). Using this model, we previously reported a robust effect of semaglutide in reducing alcohol binge-like drinking in mice (38). During the first 6 weeks, mice were given access to a sweetened alcohol solution prepared with 20% alcohol (v/v; The Warner-Graham Company), 3% glucose (w/v; dextrose; Caroline Biological Supply Company) and 0.1% saccharin (w/v; Sigma-Aldrich) 4 days a week, during alternating 2-hour (Monday and Thursday) and 4-hour (Tuesday and Friday) sessions with 1 day off (Wednesday) in between. For linagliptin testing, mice were assigned to treatment groups (linagliptin or vehicle, between-subjects) matched by sex and baseline drinking (i.e., average 4-hour session alcohol intake over the last 4 sessions prior to pharmacological testing). Linagliptin was administered on the first 4-hour test day of each week (Tuesdays) and the effects on binge-like alcohol drinking were tested (Figure 5A). For omarigliptin testing, we used a shorter and within-subjects design and tested its effect on 4-hour drinking sessions (Figure 5B). Pretreatment time was 1 hour for both linagliptin and omarigliptin. Food and water were removed from the home cages 3 hours into the dark phase and replaced with the sweetened alcohol described above (69). Alcohol intake (g/kg of body weight) was calculated from the change in weight of the drinking bottles before and after 4-hour drinking sessions.

#### Glucose-challenge tests in mice.

Given the well-known effect of DPP-4Is to lower blood glucose levels and their approved clinical use for diabetes treatment, we tested the effects of linagliptin and omarigliptin on blood glucose levels as an indirect measure of target engagement in the presence or absence of alcohol coadministration. Separate cohorts of adult C57BL/6J mice (*n* = 4 males, 4 females, per drug condition, per experiment) were fasted for approximately 4 hours and linagliptin (20 mg/kg, i.p.) or vehicle was administered in a between-subjects fashion, with treatment groups (*n* = 8) matched by body weight and sex. Thirty minutes after linagliptin administration, mice were administered (i.p.) a glucose solution (0.2 mL/25 g body weight, 20% dextrose in 0.9% saline). This experiment was repeated in a separate cohort of mice (*n* = 4 males, 4 females) with glucose plus alcohol (20% v/v, approximately 1.2–1.5 g/kg alcohol). Glucose levels were measured 45 minutes after glucose administration in blood collected via tail snip using a glucometer (HT100; Tyson Bioresearch Inc.). These experiments were repeated in separate cohorts of mice (*n* = 4 males, 4 females, per drug condition, per experiment) with omarigliptin (20 mg/kg, i.p.), following the same design.

#### Chronic, intermittent alcohol vapor exposure and operant oral alcohol self administration in rats.

Rats were made dependent on alcohol by chronic, intermittent alcohol vapor exposure (61, 70, 128). Using this model, we previously reported a robust effect of semaglutide in reducing operant oral alcohol self administration in rats (38). Rats were exposed to alcohol vapor 14 hours per day to reach blood alcohol levels of 150–250 mg/dL, followed by 10 hours of room air (alcohol withdrawal). After at least 8 weeks of alcohol vapor exposure, behavioral testing was conducted during acute spontaneous withdrawal (6–8 hours after alcohol vapor was turned off). Daily vapor exposure continued during the entire experiment.

Alcohol-dependent male (*n* = 10) and female (*n* = 9) rats were trained to self administer unsweetened oral alcohol (10% w/v, approximately 12.6% v/v) and water, as previously described (129). Operant responses to the alcohol- or water-associated levers were reinforced with the delivery of 0.1 mL of fluid. Operant responses to the alcohol-associated lever were paired with a cue light that was illuminated during alcohol delivery (2 seconds). Additional lever presses during this period did not lead to additional fluid delivery. No cue light was paired with the delivery of water. Once presses for alcohol and water were established (approximately 16 training sessions), rats underwent 30-minute, fixed ratio 1 (FR1), self-administration sessions. Vehicle, linagliptin (10, 20 mg/kg; 2.5-hour pretreatment), or omarigliptin (10, 20 mg/kg; 1.5-hour pretreatment) was administered i.p. prior to each self-administration session in a randomized (Latin-square), within-subjects fashion (Figure 5C). Tests of different doses of the same drug were separated by at least 2 days. Alcohol intake (g/kg of body weight) was calculated from the total volume of alcohol solution delivered per 30-minute operant sessions. Of note, for these experiments, we used the same rats that had been tested with semaglutide in our previous study (38). A minimum of 3 weeks of washout was employed between the previous semaglutide testing and the present DPP-4Is testing.

#### Statistics.

The mouse data with s.c. linagliptin were analyzed using 3-way repeated-measures analysis of variance (ANOVA) with drug and sex as between-subjects factors and week (corresponding to escalating linagliptin doses) as a within-subjects factor. All other rodent data were analyzed using 2-way ANOVAs. The Grubb’s test identified no significant outliers. 2-tailed *P* values <0.05 were considered statistically significant. Prism 10 (GraphPad Prism) was used for data analysis.

### Study approval

The pharmacoepidemiologic analyses were approved by the institutional review boards of Yale University and VA Connecticut Healthcare System; it was granted a waiver of informed consent and is compliant with the Health Insurance Portability and Accountability Act. The rodent studies were approved by the Institutional Animal Care and Use Committee of the National Institute on Drug Abuse Intramural Research Program.

### Data availability

Consistent with other studies based on VA data, the electronic health records are not permitted to leave the VA without a data use agreement. However, VA data are made freely available to researchers with an approved study protocol. For more information, please visit https://www.virec.research.va.gov or contact VIReC@va.gov. The rodent data and aggregate human data are available in the Supporting Data Values file.

## Author contributions

MF was responsible for conceptualization, methodology, validation, analysis, investigation, resources, data curation, writing (original draft), visualization, project administration, and funding. JT was responsible for software, analysis, data curation, and writing (review/editing). CLP was responsible for software, analysis, data curation, writing (original draft), and visualization. NB was responsible for software, analysis, data curation, writing (original draft), and visualization. JCG was responsible for writing (review/editing). VLR was responsible for writing (review/editing). DAF was responsible for writing (review/editing). HRK was responsible for writing (review/editing). GFK was responsible for validation, resources, writing (review/editing), supervision, and funding. ACJ was responsible for resources, writing (review/editing), supervision, and funding. LFV was responsible for conceptualization, methodology, software, validation, analysis, investigation, resources, writing (review/editing), visualization, supervision, project administration, and funding. CTR was responsible for conceptualization, methodology, software, validation, analysis, investigation, resources, data curation, writing (original draft), visualization, supervision, project administration, and funding. LL was responsible for conceptualization, methodology, validation, investigation, resources, writing (review/editing), supervision, project administration, and funding.

## Supplementary Material

Supplemental data

ICMJE disclosure forms

Supporting data values

## Figures and Tables

**Table 1:**
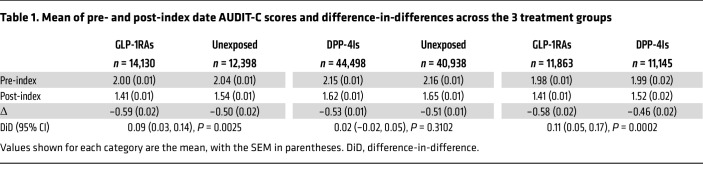
Mean of pre- and post-index date AUDIT-C scores and difference-in-differences across the 3 treatment groups

## Version 1 03/06/2025

In-Press Preview

## Version 2 05/01/2025

Electronic publication
