# Supplementary material for: Glucagon-like peptide-1 receptor agonists, but not dipeptidyl peptidase-4 inhibitors, reduce alcohol intake
Source: J Clin Invest. 2025 Mar 6;135(9):e188314. doi: 10.1172/JCI188314 (PMC12043080; doi:10.1172/JCI188314)
Supplement: Supplemental data [file jci-135-188314-s215.pdf]

Supplementary Figures

- **Figure S1.** Distribution of propensity scores for each exposure contrast before and after matching.
- **Figure S2.** Association between receipt of the glucagon-like peptide-1 receptor agonist (GLP-1RA) semaglutide and alcohol use in humans.
- **Figure S3.** Effect of the dipeptidyl peptidase-4 inhibitor (DPP-4I) linagliptin (repeated subcutaneous injections) on alcohol intake in mice.
- **Figure S4.** Effect of the dipeptidyl peptidase-4 inhibitor (DPP-4I) linagliptin (repeated subcutaneous injections) on body weight in mice.
- **Figure S5.** Effect of the dipeptidyl peptidase-4 inhibitor (DPP-4I) linagliptin (single intraperitoneal injection) on alcohol intake in mice.
- **Figure S6.** Effects of the dipeptidyl peptidase-4 inhibitors (DPP-4Is) linaglipin and omarigliptin on alcohol deliveries and water intake in rats.

Figure S1. Distribution of propensity scores for each exposure contrast before and after matching.

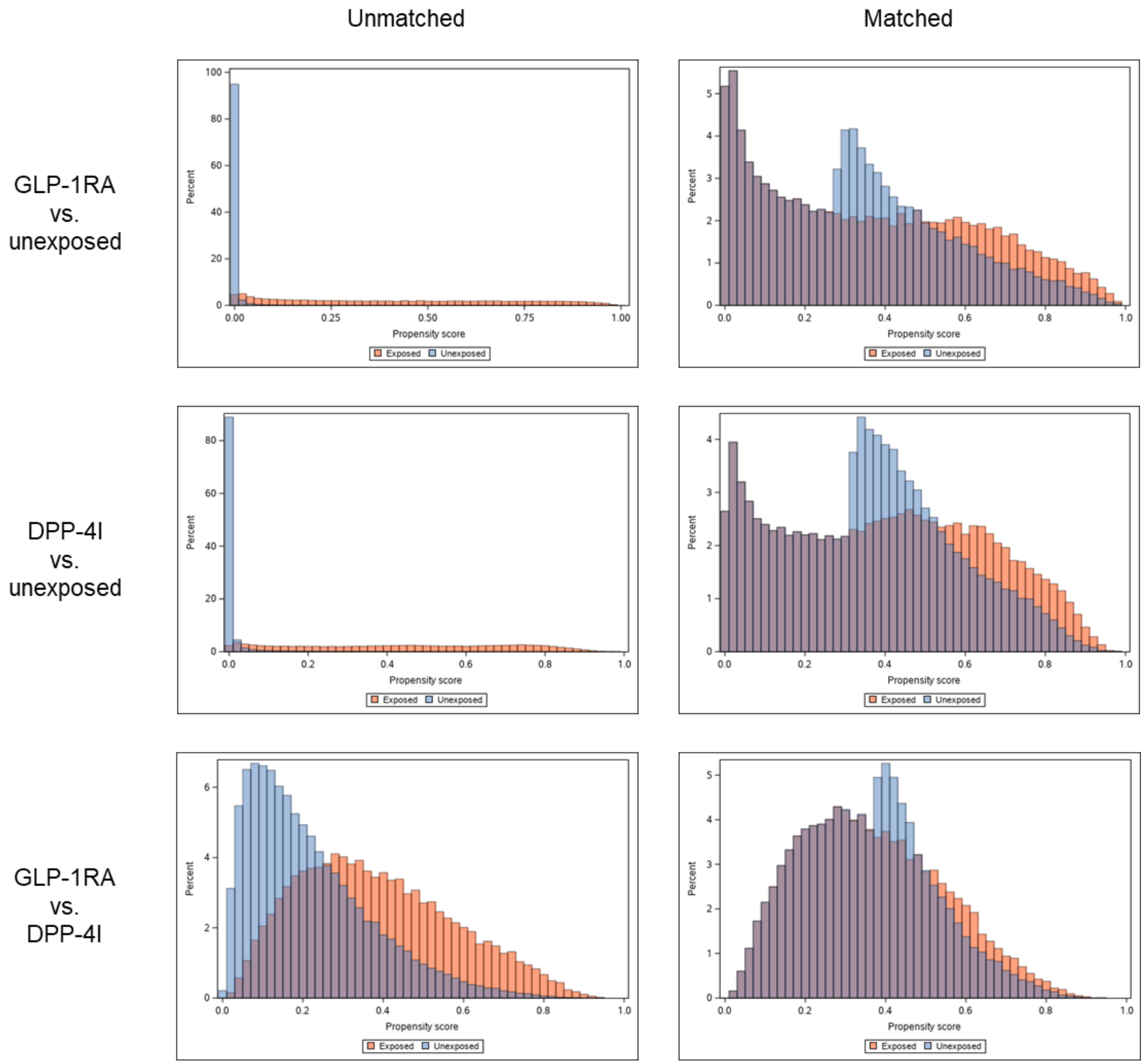

Figure S2. Association between receipt of the glucagon-like peptide-1 receptor agonist (GLP-1RA) semaglutide and alcohol use in humans. Difference-in-difference estimates and 95% confidence intervals of changes in Alcohol Use Disorders Identification Test-Consumption-C (AUDIT-C) scores, overall (white) and stratified by baseline AUD diagnosis (green) and by baseline AUDIT-C score (blue). (A) Semaglutide recipients vs. unexposed individuals, (B) Semaglutide recipients vs. dipeptidyl peptidase-4 inhibitors (DPP-4Is) recipients. **p* < 0.05, ***p < 0.001, *****p* < 0.0001, *NS* not significant.

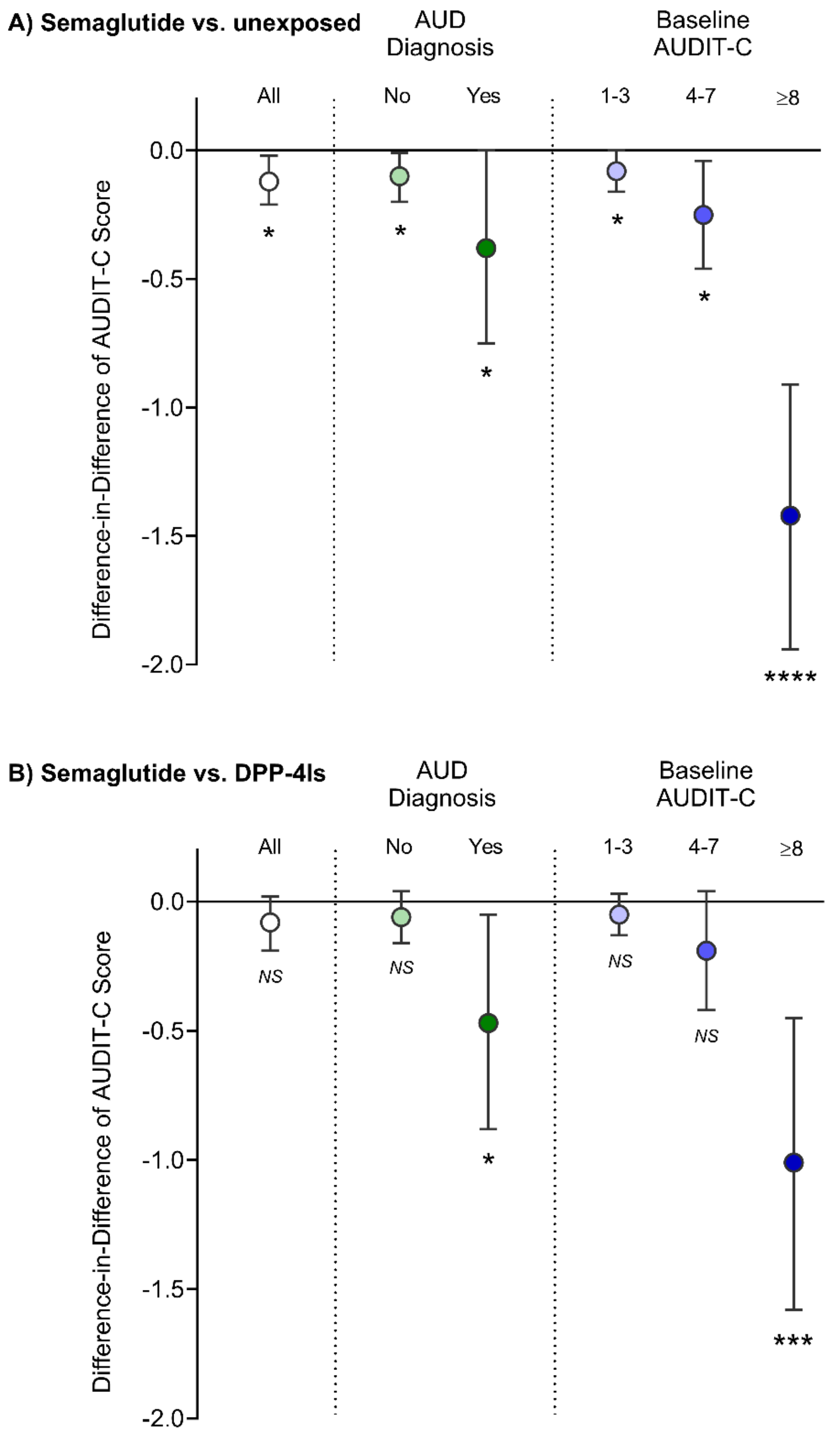

Figure S3. Effect of the dipeptidyl peptidase-4 inhibitor (DPP-4I) linagliptin (repeated subcutaneous injections) on alcohol intake in mice. Linagliptin (2.5, 5, 10, and 20 mg/kg, s.c.), tested using a between-subjects design, had no effect on binge-like alcohol drinking, measured on the second 4-h session (Friday) each week, in mice (*n* = 16 males, 16 females). Drug (linagliptin) effect: $F_{1,28} = 0.78$, $p = 0.38$; Week effect: $F_{3,84} = 7.23$, $p = 0.0002$ (week 4 < weeks 1, 2, and 3); Sex effect: $F_{1,28} = 0.07$, $p = 0.79$; Drug × Week interaction: $F_{3,84} = 1.94$, $p = 0.13$; Drug × Sex interaction: $F_{1,28} = 0.004$, $p = 0.95$; Week × Sex interaction: $F_{3,84} = 1.70$, $p = 0.17$; Drug × Week × Sex interaction: $F_{3,84} = 1.33$, $p = 0.27$. Individual data symbols are shown in black for males and in gray for females. Data are expressed as mean (standard error).

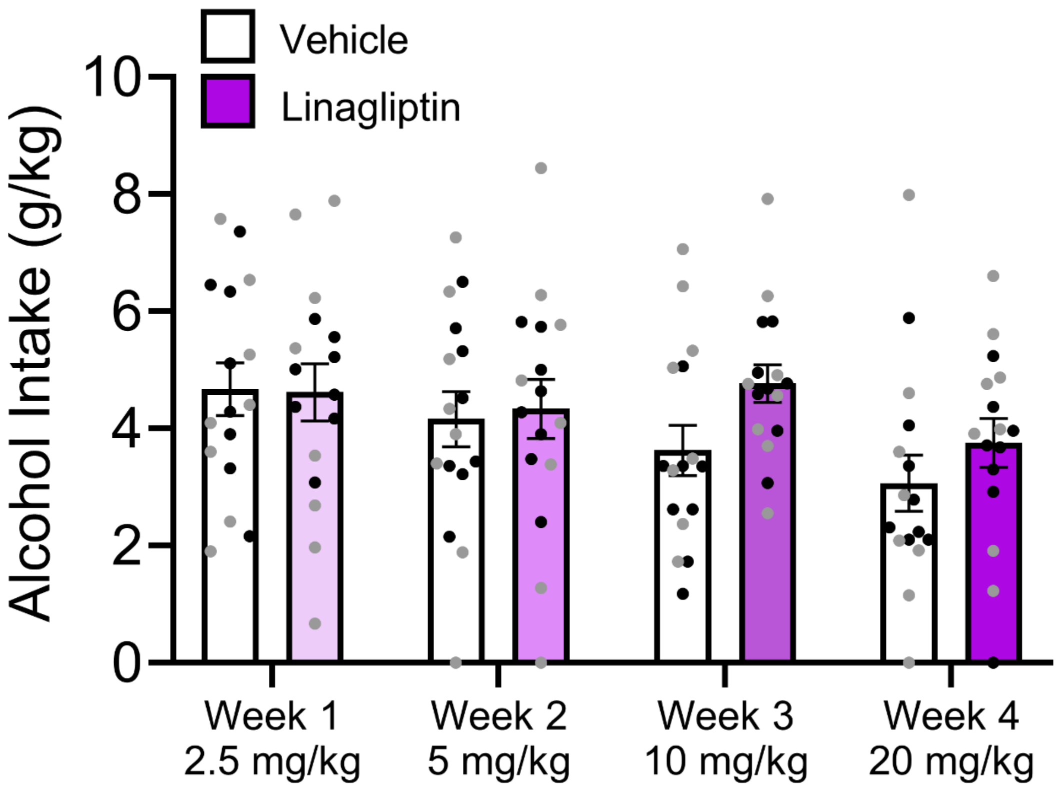

Figure S4. Effect of the dipeptidyl peptidase-4 inhibitor (DPP-4I) linagliptin (repeated subcutaneous injections) on body weight in mice. Linagliptin (2.5, 5, 10, and 20 mg/kg, s.c.), tested using a between-subjects design, had no effect on body weight in mice (*n* = 16 males, 16 females). Drug (linagliptin) effect: $F_{1,28} = 0.51$, $p = 0.48$; Week effect: $F_{4,112} = 79.68$, $p < 0.0001$ (baseline < weeks 1, 2, 3, and 4; week 1 < weeks 3 and 4; week 2 < weeks 3 and 4); Sex effect: $F_{1,28} = 71.65$, $p < 0.0001$ (male > female); Drug × Week interaction: $F_{4,112} = 0.32$, $p = 0.87$; Drug × Sex interaction: $F_{1,28} = 0.18$, $p = 0.67$; Week × Sex interaction: $F_{4,112} = 1.23$, $p = 0.30$; Drug × Week × Sex interaction: $F_{4,112} = 1.48$, $p = 0.21$. Individual data symbols are shown in black for males and in gray for females. Data are expressed as mean (standard error).

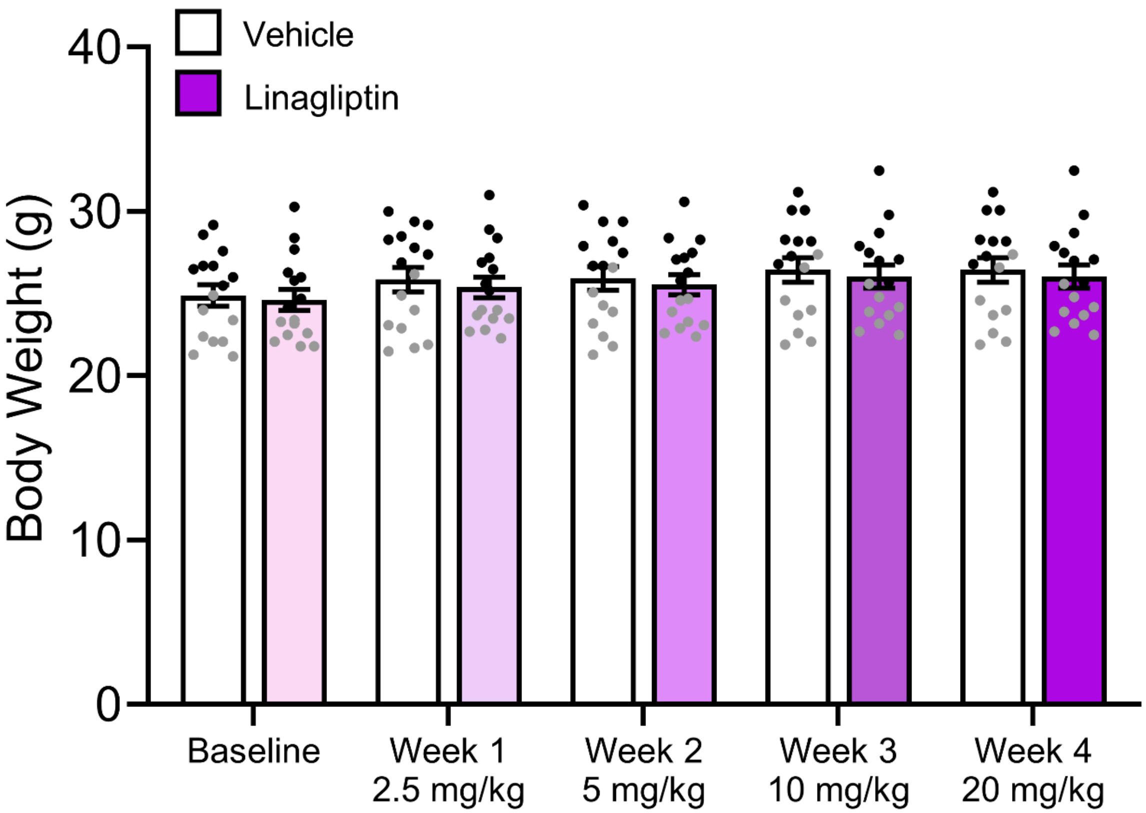

Figure S5. Effect of the dipeptidyl peptidase-4 inhibitor (DPP-4I) linagliptin (single intraperitoneal injection) on alcohol intake in mice. Linagliptin (20 mg/kg, i.p.), tested using a between-subjects design, had no effect on binge-like alcohol drinking in mice (*n* = 16 males, 16 females). Drug (linagliptin) effect: $F_{1,14} = 0.50$, $p = 0.49$; Sex effect: $F_{1,14} = 0.55$, $p = 0.47$; Drug × Sex interaction: $F_{1,14} = 0.09$, $p = 0.77$. Individual data symbols are shown in black for males and in gray for females. Data are expressed as mean (standard error).

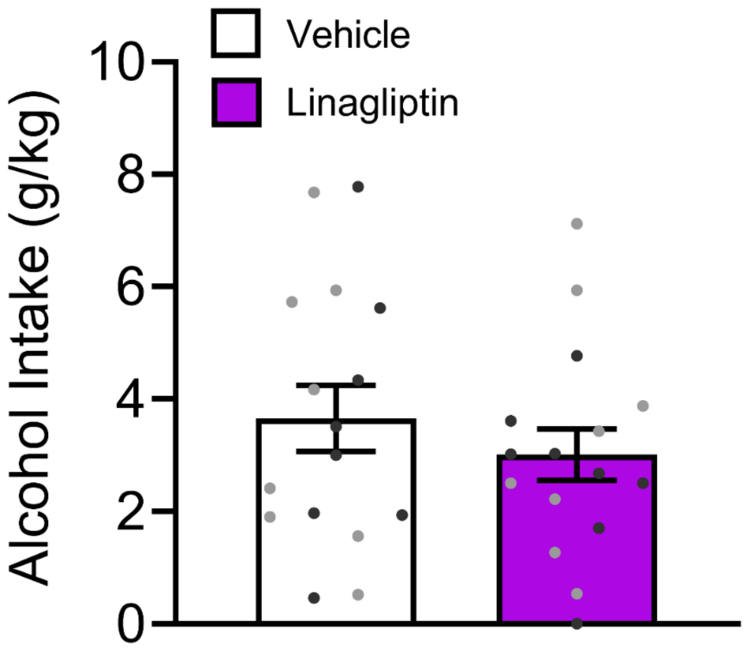

Figure S6. Effects of the dipeptidyl peptidase-4 inhibitors (DPP-4Is) linagliptin and omarigliptin on alcohol deliveries and water intake in rats. (**A**) Linagliptin (10, 20 mg/kg, i.p.), tested using a within-subjects design, had no effect on number of alcohol deliveries during operant oral alcohol self-administration in alcohol-dependent rats (*n* = 10 males, 9 females). Drug (linagliptin) effect: $F_{2,34} = 0.34$, $p = 0.72$; Sex effect: $F_{1,17} = 15.78$, $p = 0.001$ (male > female); Drug × Sex interaction: $F_{2,34} = 2.94$, $p = 0.07$. (**B**) Linagliptin (10, 20 mg/kg, i.p.), tested using a within-subjects design, had no effect on water intake in alcohol-dependent rats. Drug (linagliptin) effect: $F_{2,34} = 1.55$, $p = 0.23$; Sex effect: $F_{1,17} = 3.53$, $p = 0.08$; Drug × Sex interaction: $F_{2,34} = 1.47$, $p = 0.24$. (**C**) Omarigliptin (10, 20 mg/kg, i.p.), tested using a within-subjects design, had no effect on number of alcohol deliveries during operant oral alcohol self-administration in alcohol-dependent rats (*n* = 10 males, 9 females). Drug (omarigliptin) effect: $F_{2,34} = 1.37$, $p = 0.27$; Sex effect: $F_{1,17} = 5.16$, $p = 0.04$ (male > female); Drug × Sex interaction: $F_{2,34} = 0.09$, $p = 0.91$. (**D**) Omarigliptin (10, 20 mg/kg, i.p.), tested using a within-subjects design, had no effect on water intake in alcohol-dependent rats. Drug (omarigliptin) effect: $F_{2,34} = 0.04$, $p = 0.96$; Sex effect: $F_{1,17} = 0.61$, $p = 0.45$; Drug × Sex interaction: $F_{2,34} = 2.43$, $p = 0.10$. Individual data symbols are shown in black for males and in gray for females. Data are expressed as mean (standard error).

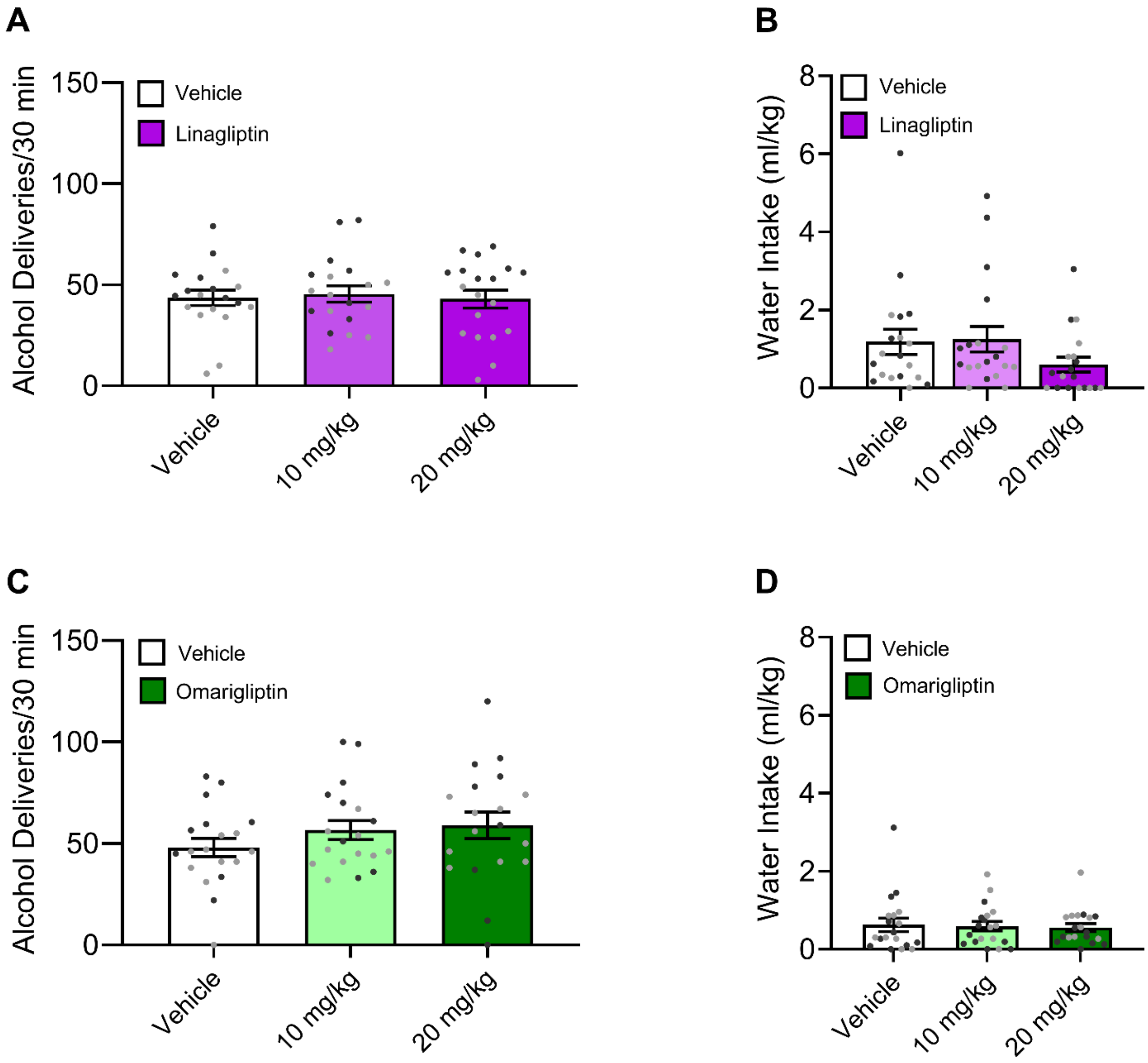

Supplementary Tables

- **Table S1.** Characteristics between glucagon-like peptide-1 receptor agonist (GLP-1RA) recipients and unexposed individuals, before and after propensity score matching and recorded follow-up alcohol consumption.

- **Table S2.** Characteristics between dipeptidyl-peptidase 4 inhibitor (DPP-4I) recipients and unexposed individuals, before and after propensity score matching and recorded follow-up alcohol consumption.

- **Table S3.** Characteristics between glucagon-like peptide-1 receptor agonist (GLP-1RA) and dipeptidyl-peptidase 4 inhibitor (DPP-4I) recipients, before and after propensity score matching and recorded follow-up alcohol consumption.

- **Table S4.** Mean (standard error) of pre- and post-index date AUDIT-C scores and difference-in-differences (DiD) across the three treatment groups, stratified by baseline AUD diagnosis.

- **Table S5.** Mean (standard error) of pre- and post-index date AUDIT-C scores and difference-in-differences (DiD) across the three treatment groups, stratified by baseline AUDIT-C score.

- **Table S6.** Mean (standard error) of pre- and post-index date AUDIT-C scores and difference-in-differences (DiD) across the three treatment groups, stratified by baseline BMI.

- **Table S7.** Mean (standard error) of pre- and post-index date AUDIT-C scores and difference-in-differences (DiD) among semaglutide recipients and propensity score-matched unexposed comparators and DPP-4Is, overall and stratified by baseline AUD diagnosis, baseline AUDIT-C score, and baseline BMI.

Table S1. Characteristics between glucagon-like peptide-1 receptor agonist (GLP-1RA) recipients and unexposed individuals, before and after propensity score matching and recorded follow-up alcohol consumption.

	**Unmatched**			**Matched**			**Matched with follow-up AUDIT-C**		
	**GLP-1RA**	**Unexposed**	**SMD**	**GLP-1RA**	**Unexposed**	**SMD**	**GLP-1RA**	**Unexposed**	**SMD**
Sample size, n	30329	3397092		27231	27231		14130	12398	
**Demographics**									
Age, years									
20-49	3990 (13.2)	1084052 (31.9)	0.46	3590 (13.2)	3338 (12.3)	0.028	1625 (11.5)	1395 (11.3)	0.008
50-59	6569 (21.7)	510665 (15.0)	0.17	5687 (20.9)	5584 (20.5)	0.009	2849 (20.2)	2514 (20.3)	0.003
60-69	10132 (33.4)	829730 (24.4)	0.20	9150 (33.6)	9309 (34.2)	0.012	4856 (34.4)	4374 (35.3)	0.019
70-74	6633 (21.9)	346176 (10.2)	0.32	5958 (21.9)	5977 (21.9)	0.002	3317 (23.5)	2795 (22.5)	0.022
75-79	2134 (7.0)	234643 (6.9)	0.01	1986 (7.3)	2088 (7.7)	0.014	1043 (7.4)	931 (7.5)	0.005
≥80	871 (2.9)	391826 (11.5)	0.35	860 (3.2)	935 (3.4)	0.015	440 (3.1)	389 (3.1)	0.001
Race/ethnicity									
White	21462 (70.8)	2356397 (69.4)	0.03	19184 (70.4)	19099 (70.1)	0.007	10045 (71.1)	8649 (69.8)	0.029
Black	4585 (15.1)	486723 (14.3)	0.02	4186 (15.4)	4276 (15.7)	0.009	2076 (14.7)	1989 (16.0)	0.037
Hispanic	2284 (7.5)	219207 (6.5)	0.04	2044 (7.5)	2053 (7.5)	0.001	1077 (7.6)	944 (7.6)	0.000
Asian	226 (0.7)	33597 (1.0)	0.03	209 (0.8)	201 (0.7)	0.003	112 (0.8)	91 (0.7)	0.007
AI/AN	198 (0.7)	19995 (0.6)	0.01	178 (0.7)	188 (0.7)	0.004	87 (0.6)	85 (0.7)	0.009
PI/NH	265 (0.9)	22349 (0.7)	0.02	237 (0.9)	246 (0.9)	0.004	114 (0.8)	107 (0.9)	0.006
Mixed race	227 (0.7)	25790 (0.8)	0.00	201 (0.7)	189 (0.7)	0.005	107 (0.8)	84 (0.7)	0.009
Other/unknown	1082 (3.6)	233034 (6.9)	0.15	992 (3.6)	979 (3.6)	0.003	512 (3.6)	449 (3.6)	0.000
Sex									
Female	2201 (7.3)	250487 (7.4)	0.00	1899 (7.0)	1851 (6.8)	0.007	919 (6.5)	870 (7.0)	0.020
Male	28128 (92.7)	3146605 (92.6)	0.00	25332 (93.0)	25380 (93.2)	0.007	13211 (93.5)	11528 (93.0)	0.020
Urban residence	19332 (63.7)	2265441 (66.7)	0.06	17379 (63.8)	17425 (64.0)	0.004	8959 (63.4)	7909 (63.8)	0.008
Census region									
Midwest	7947 (26.2)	758021 (22.3)	0.09	7138 (26.2)	7178 (26.4)	0.003	3630 (25.7)	3240 (26.1)	0.010
Northeast	4134 (13.6)	479431 (14.1)	0.01	3636 (13.4)	3578 (13.1)	0.006	1932 (13.7)	1659 (13.4)	0.009
South	11444 (37.7)	1392728 (41.0)	0.07	10374 (38.1)	10431 (38.3)	0.004	5525 (39.1)	4929 (39.8)	0.013
West	6804 (22.4)	766912 (22.6)	0.00	6083 (22.3)	6044 (22.2)	0.003	3043 (21.5)	2570 (20.7)	0.020
Year of index date									

2009-2012	690 (2.3)	1009701 (29.7)	0.85	690 (2.5)	694 (2.5)	0.001	340 (2.4)	329 (2.7)	0.016
2013-2015	1569 (5.2)	780170 (23.0)	0.54	1554 (5.7)	1710 (6.3)	0.024	767 (5.4)	820 (6.6)	0.050
2016-2018	10363 (34.2)	831213 (24.5)	0.21	9437 (34.7)	9584 (35.2)	0.011	5054 (35.8)	4593 (37.0)	0.027
2019-2021	17707 (58.4)	776008 (22.8)	0.74	15550 (57.1)	15243 (56.0)	0.023	7969 (56.4)	6656 (53.7)	0.055
**Clinical characteristics**									
Alcohol use disorder	1762 (5.8)	357445 (10.5)	0.17	1657 (6.1)	1657 (6.1)	0.000	881 (6.2)	743 (6.0)	0.010
Asthma	1202 (4.0)	67082 (2.0)	0.12	1035 (3.8)	1016 (3.7)	0.004	562 (4.0)	521 (4.2)	0.011
Bariatric surgery	52 (0.2)	1422 (0.0)	0.04	40 (0.1)	48 (0.2)	0.007	21 (0.1)	28 (0.2)	0.018
Cancer, localized	2185 (7.2)	240283 (7.1)	0.01	1997 (7.3)	2041 (7.5)	0.006	1082 (7.7)	1031 (8.3)	0.024
Cancer, metastatic	106 (0.3)	20538 (0.6)	0.04	102 (0.4)	100 (0.4)	0.001	50 (0.4)	40 (0.3)	0.005
Cerebrovascular disease	2350 (7.7)	135859 (4.0)	0.16	2120 (7.8)	2072 (7.6)	0.007	1159 (8.2)	1002 (8.1)	0.004
COPD	5645 (18.6)	414178 (12.2)	0.18	5035 (18.5)	4943 (18.2)	0.009	2749 (19.5)	2454 (19.8)	0.009
Congestive heart failure	3844 (12.7)	118003 (3.5)	0.35	3394 (12.5)	3375 (12.4)	0.002	1898 (13.4)	1705 (13.8)	0.009
Dementia	381 (1.3)	26537 (0.8)	0.05	355 (1.3)	369 (1.4)	0.004	185 (1.3)	168 (1.4)	0.004
Diabetes, complications	16083 (53.0)	121493 (3.6)	1.25	13785 (50.6)	13454 (49.4)	0.024	7396 (52.3)	6522 (52.6)	0.005
Diabetes, w/o complications	28386 (93.6)	456500 (13.4)	1.88	25290 (92.9)	25610 (94.0)	0.048	13326 (94.3)	11773 (95.0)	0.029
Hemiplegia or paraplegia	160 (0.5)	14848 (0.4)	0.01	153 (0.6)	155 (0.6)	0.001	91 (0.6)	72 (0.6)	0.008
HIV	140 (0.5)	11533 (0.3)	0.02	121 (0.4)	126 (0.5)	0.003	67 (0.5)	77 (0.6)	0.020
Liver disease, mild	2335 (7.7)	112787 (3.3)	0.20	2055 (7.5)	1945 (7.1)	0.015	1146 (8.1)	923 (7.4)	0.025
Liver disease, moderate to severe	126 (0.4)	12700 (0.4)	0.01	117 (0.4)	117 (0.4)	0.000	68 (0.5)	53 (0.4)	0.008
Myocardial infarction	1423 (4.7)	50430 (1.5)	0.19	1264 (4.6)	1217 (4.5)	0.008	682 (4.8)	613 (4.9)	0.005
Opioid use disorder	206 (0.7)	32127 (0.9)	0.03	185 (0.7)	192 (0.7)	0.003	112 (0.8)	92 (0.7)	0.006
Peptic ulcer disease	223 (0.7)	24291 (0.7)	0.00	198 (0.7)	191 (0.7)	0.003	111 (0.8)	100 (0.8)	0.002
Peripheral vascular disease	3490 (11.5)	160397 (4.7)	0.25	3128 (11.5)	3085 (11.3)	0.005	1754 (12.4)	1541 (12.4)	0.000
PTSD	5027 (16.6)	352866 (10.4)	0.18	4400 (16.2)	4303 (15.8)	0.010	2361 (16.7)	2215 (17.9)	0.031
Renal disease	5350 (17.6)	145280 (4.3)	0.45	4619 (17.0)	4651 (17.1)	0.003	2560 (18.1)	2298 (18.5)	0.011
Rheumatic disease	414 (1.4)	33368 (1.0)	0.04	375 (1.4)	366 (1.3)	0.003	203 (1.4)	179 (1.4)	0.001
Charlson Comorbidity Index									
0	1048 (3.5)	2167254 (63.8)	1.48	1048 (3.8)	806 (3.0)	0.049	402 (2.8)	277 (2.2)	0.039
1	7818 (25.8)	564951 (16.6)	0.22	7331 (26.9)	7727 (28.4)	0.033	3644 (25.8)	3174 (25.6)	0.004
2	8623 (28.4)	326179 (9.6)	0.49	7623 (28.0)	7525 (27.6)	0.008	3961 (28.0)	3445 (27.8)	0.005
3	4566 (15.1)	157219 (4.6)	0.36	4035 (14.8)	4075 (15.0)	0.004	2139 (15.1)	1927 (15.5)	0.011

4	3567 (11.8)	79011 (2.3)	0.39	3102 (11.4)	3052 (11.2)	0.006	1637 (11.6)	1487 (12.0)	0.013
≥5	4707 (15.5)	102478 (3.0)	0.46	4092 (15.0)	4046 (14.9)	0.005	2347 (16.6)	2088 (16.8)	0.006
VACS Index									
≤54	2709 (8.9)	665769 (19.6)	0.31	2440 (9.0)	2335 (8.6)	0.014	1120 (7.9)	1010 (8.1)	0.008
>54-61	4975 (16.4)	450956 (13.3)	0.09	4416 (16.2)	4446 (16.3)	0.003	2186 (15.5)	1988 (16.0)	0.015
>61-74	10867 (35.8)	763041 (22.5)	0.30	9666 (35.5)	9623 (35.3)	0.003	5132 (36.3)	4473 (36.1)	0.005
>74	7271 (24.0)	633207 (18.6)	0.13	6597 (24.2)	6813 (25.0)	0.018	3609 (25.5)	3259 (26.3)	0.017
Missing	4507 (14.9)	884119 (26.0)	0.28	4112 (15.1)	4014 (14.7)	0.010	2083 (14.7)	1668 (13.5)	0.037
**Other medications at index date**									
Any alcohol pharmacotherapy	7083 (23.4)	183186 (5.4)	0.54	6120 (22.5)	5955 (21.9)	0.015	3306 (23.4)	2894 (23.3)	0.001
Any neurocognitively-active	15455 (51.0)	864340 (25.4)	0.53	13568 (49.8)	13318 (48.9)	0.018	7255 (51.3)	6473 (52.2)	0.017
Any anticholinergic	6647 (21.9)	313852 (9.2)	0.36	5854 (21.5)	5791 (21.3)	0.006	3215 (22.8)	2833 (22.9)	0.002
Medication count									
0-1	912 (3.0)	2165621 (63.7)	1.50	912 (3.3)	714 (2.6)	0.043	368 (2.6)	215 (1.7)	0.060
2-4	4800 (15.8)	785028 (23.1)	0.18	4726 (17.4)	5412 (19.9)	0.065	2257 (16.0)	2163 (17.4)	0.040
5-9	15320 (50.5)	395670 (11.6)	0.88	14103 (51.8)	14528 (53.4)	0.031	7287 (51.6)	6723 (54.2)	0.053
≥10	9297 (30.7)	50773 (1.5)	0.93	7490 (27.5)	6577 (24.2)	0.077	4218 (29.9)	3297 (26.6)	0.072
**Substance use**									
Alcohol consumption (AUDIT-C score)									
Low-risk (1-3)	26548 (87.5)	2413092 (71.0)	0.41	23664 (86.9)	23664 (86.9)	0.000	12206 (86.4)	10698 (86.3)	0.003
At-risk (4-7)	3296 (10.9)	787312 (23.2)	0.33	3087 (11.3)	3087 (11.3)	0.000	1677 (11.9)	1480 (11.9)	0.002
Hazardous/binge (≥8)	485 (1.6)	196688 (5.8)	0.23	480 (1.8)	480 (1.8)	0.000	247 (1.7)	220 (1.8)	0.002
Substance use treatment program visit	1431 (4.7)	320642 (9.4)	0.19	1349 (5.0)	1353 (5.0)	0.001	717 (5.1)	624 (5.0)	0.002
Smoking status									
Never	9432 (31.1)	1035672 (30.5)	0.01	8393 (30.8)	8323 (30.6)	0.006	4252 (30.1)	3681 (29.7)	0.009
Former	12792 (42.2)	1090452 (32.1)	0.21	11443 (42.0)	11551 (42.4)	0.008	6096 (43.1)	5309 (42.8)	0.006
Current	8033 (26.5)	1207183 (35.5)	0.20	7325 (26.9)	7294 (26.8)	0.003	3760 (26.6)	3389 (27.3)	0.016
Missing	72 (0.2)	63785 (1.9)	0.18	70 (0.3)	63 (0.2)	0.005	22 (0.2)	19 (0.2)	0.001
**Vital signs**									
Body mass index, kg/m^2									

≤26	1151 (3.8)	942912 (27.8)	0.72	1138 (4.2)	1239 (4.5)	0.018	543 (3.8)	544 (4.4)	0.027
>26-32	7557 (24.9)	1411064 (41.5)	0.36	7153 (26.3)	7475 (27.5)	0.027	3647 (25.8)	3381 (27.3)	0.033
>32	20633 (68.0)	855765 (25.2)	0.89	17995 (66.1)	17579 (64.6)	0.032	9483 (67.1)	8119 (65.5)	0.034
Missing	988 (3.3)	187351 (5.5)	0.11	945 (3.5)	938 (3.4)	0.001	457 (3.2)	354 (2.9)	0.022
Systolic blood pressure, mm Hg									
≤127	9496 (31.3)	1130585 (33.3)	0.04	8472 (31.1)	8398 (30.8)	0.006	4413 (31.2)	3847 (31.0)	0.004
>127-141	12006 (39.6)	1098716 (32.3)	0.15	10728 (39.4)	10772 (39.6)	0.003	5573 (39.4)	4961 (40.0)	0.012
>141	8411 (27.7)	657807 (19.4)	0.20	7628 (28.0)	7773 (28.5)	0.012	3993 (28.3)	3509 (28.3)	0.001
Missing	416 (1.4)	509984 (15.0)	0.56	403 (1.5)	288 (1.1)	0.038	151 (1.1)	81 (0.7)	0.045
Diastolic blood pressure, mm Hg									
≤82	22296 (73.5)	1999683 (58.9)	0.31	19924 (73.2)	19972 (73.3)	0.004	10577 (74.9)	9270 (74.8)	0.002
>82	7617 (25.1)	887425 (26.1)	0.02	6904 (25.4)	6971 (25.6)	0.006	3402 (24.1)	3047 (24.6)	0.012
Missing	416 (1.4)	509984 (15.0)	0.56	403 (1.5)	288 (1.1)	0.038	151 (1.1)	81 (0.7)	0.045
**Laboratory findings**									
Albumin, g/dL									
≤4.0	14223 (46.9)	1043584 (30.7)	0.33	12677 (46.6)	12701 (46.6)	0.002	6644 (47.0)	5985 (48.3)	0.025
>4.0-4.4	9348 (30.8)	1002544 (29.5)	0.03	8362 (30.7)	8403 (30.9)	0.003	4329 (30.6)	3807 (30.7)	0.002
>4.4	3592 (11.8)	549060 (16.2)	0.12	3260 (12.0)	3296 (12.1)	0.004	1699 (12.0)	1424 (11.5)	0.017
Missing	3166 (10.4)	801904 (23.6)	0.36	2932 (10.8)	2831 (10.4)	0.012	1458 (10.3)	1182 (9.5)	0.026
Total cholesterol, mg/dL									
≤137	10059 (33.2)	398730 (11.7)	0.53	8881 (32.6)	9044 (33.2)	0.013	4872 (34.5)	4276 (34.5)	0.000
>137-220	16132 (53.2)	1940768 (57.1)	0.08	14532 (53.4)	14425 (53.0)	0.008	7496 (53.1)	6583 (53.1)	0.001
>220	2454 (8.1)	422070 (12.4)	0.14	2246 (8.2)	2303 (8.5)	0.008	1024 (7.2)	969 (7.8)	0.022
Missing	1684 (5.6)	635524 (18.7)	0.42	1572 (5.8)	1459 (5.4)	0.018	738 (5.2)	570 (4.6)	0.029
HDL cholesterol, mg/dL									
<35	11190 (36.9)	476928 (14.0)	0.54	9786 (35.9)	9616 (35.3)	0.013	5172 (36.6)	4437 (35.8)	0.017
>35-45	10763 (35.5)	860018 (25.3)	0.22	9656 (35.5)	9686 (35.6)	0.002	5071 (35.9)	4452 (35.9)	0.000
>45	6661 (22.0)	1416071 (41.7)	0.43	6188 (22.7)	6431 (23.6)	0.021	3134 (22.2)	2918 (23.5)	0.032
Missing	1715 (5.7)	644075 (19.0)	0.42	1601 (5.9)	1498 (5.5)	0.016	753 (5.3)	591 (4.8)	0.026
HbA1c, %									
<6.5	2048 (6.8)	1478790 (43.5)	0.92	2047 (7.5)	2233 (8.2)	0.025	979 (6.9)	1022 (8.2)	0.050
6.5-<8.0	6593 (21.7)	208018 (6.1)	0.47	6462 (23.7)	7622 (28.0)	0.097	3614 (25.6)	3598 (29.0)	0.077

≥8.0	17454 (57.5)	83437 (2.5)	1.41	14577 (53.5)	13275 (48.7)	0.096	7491 (53.0)	6018 (48.5)	0.090
Missing	4234 (14.0)	1626847 (47.9)	0.76	4145 (15.2)	4101 (15.1)	0.005	2046 (14.5)	1760 (14.2)	0.008
Hemoglobin, g/dL									
≤13.5	8896 (29.3)	643324 (18.9)	0.24	8010 (29.4)	8289 (30.4)	0.022	4312 (30.5)	3952 (31.9)	0.029
>13.5-14.3	5695 (18.8)	496410 (14.6)	0.11	5097 (18.7)	5195 (19.1)	0.009	2673 (18.9)	2404 (19.4)	0.012
>14.3	13431 (44.3)	1653052 (48.7)	0.09	12011 (44.1)	11725 (43.1)	0.021	6120 (43.3)	5233 (42.2)	0.022
Missing	2307 (7.6)	604306 (17.8)	0.31	2113 (7.8)	2022 (7.4)	0.013	1025 (7.3)	809 (6.5)	0.029
Total bilirubin, mg/dL									
≤0.6	17664 (58.2)	1358736 (40.0)	0.37	15646 (57.5)	15602 (57.3)	0.003	8121 (57.5)	7236 (58.4)	0.018
>0.6	9673 (31.9)	1290881 (38.0)	0.13	8837 (32.5)	8958 (32.9)	0.009	4647 (32.9)	4050 (32.7)	0.005
Missing	2992 (9.9)	747475 (22.0)	0.34	2748 (10.1)	2671 (9.8)	0.009	1362 (9.6)	1112 (9.0)	0.023
Triglycerides, mg/dL									
≤118	7386 (24.4)	1448094 (42.6)	0.39	6889 (25.3)	7244 (26.6)	0.030	3563 (25.2)	3358 (27.1)	0.043
>118-184	8445 (27.8)	724795 (21.3)	0.15	7621 (28.0)	7743 (28.4)	0.010	3992 (28.3)	3531 (28.5)	0.005
>184	12713 (41.9)	561442 (16.5)	0.57	11053 (40.6)	10685 (39.2)	0.028	5780 (40.9)	4888 (39.4)	0.030
Missing	1785 (5.9)	662761 (19.5)	0.42	1668 (6.1)	1559 (5.7)	0.017	795 (5.6)	621 (5.0)	0.028
White blood cell count, K/µL									
≤5.7	4811 (15.9)	832569 (24.5)	0.22	4420 (16.2)	4524 (16.6)	0.010	2267 (16.0)	2110 (17.0)	0.026
>5.7-7.2	8300 (27.4)	887653 (26.1)	0.03	7446 (27.3)	7512 (27.6)	0.005	3900 (27.6)	3471 (28.0)	0.009
>7.2	14873 (49.0)	1069377 (31.5)	0.36	13215 (48.5)	13139 (48.3)	0.006	6923 (49.0)	5992 (48.3)	0.013
Missing	2345 (7.7)	607493 (17.9)	0.31	2150 (7.9)	2056 (7.6)	0.013	1040 (7.4)	825 (6.7)	0.028
Fibrosis-4 score									
<1.45	17008 (56.1)	1529478 (45.0)	0.22	15063 (55.3)	14973 (55.0)	0.007	7697 (54.5)	6860 (55.3)	0.017
1.45-3.25	8080 (26.6)	852004 (25.1)	0.04	7375 (27.1)	7545 (27.7)	0.014	3980 (28.2)	3515 (28.4)	0.004
>3.25	676 (2.2)	129749 (3.8)	0.09	636 (2.3)	662 (2.4)	0.006	364 (2.6)	328 (2.6)	0.004
Missing	4565 (15.1)	885861 (26.1)	0.28	4157 (15.3)	4051 (14.9)	0.011	2089 (14.8)	1695 (13.7)	0.032
eGFR, mL/min									
≥60	20228 (66.7)	2444298 (72.0)	0.11	18358 (67.4)	18509 (68.0)	0.012	9411 (66.6)	8367 (67.5)	0.019
30-59	6867 (22.6)	346251 (10.2)	0.34	6032 (22.2)	6092 (22.4)	0.005	3295 (23.3)	2893 (23.3)	0.000
<30	685 (2.3)	34188 (1.0)	0.10	628 (2.3)	692 (2.5)	0.015	351 (2.5)	349 (2.8)	0.021
Missing	2549 (8.4)	572355 (16.8)	0.26	2213 (8.1)	1938 (7.1)	0.038	1073 (7.6)	789 (6.4)	0.048
**Utilization**									
Index visit in primary care	22374 (73.8)	3247031 (95.6)	0.65	20809 (76.4)	21138 (77.6)	0.029	10841 (76.7)	9235 (74.5)	0.052

No. visits to prescribing clinic in previous 2 years									
0	293 (1.0)	554547 (16.3)	0.63	287 (1.1)	186 (0.7)	0.040	109 (0.8)	47 (0.4)	0.053
1-2	1363 (4.5)	787751 (23.2)	0.58	1329 (4.9)	1420 (5.2)	0.015	539 (3.8)	422 (3.4)	0.022
3-4	1823 (6.0)	612358 (18.0)	0.38	1772 (6.5)	2048 (7.5)	0.040	871 (6.2)	743 (6.0)	0.007
5-6	2144 (7.1)	419901 (12.4)	0.18	2077 (7.6)	2231 (8.2)	0.021	1033 (7.3)	893 (7.2)	0.004
7-12	6874 (22.7)	609022 (17.9)	0.12	6427 (23.6)	6697 (24.6)	0.023	3248 (23.0)	2887 (23.3)	0.007
≥12	17832 (58.8)	413513 (12.2)	1.03	15339 (56.3)	14649 (53.8)	0.051	8330 (59.0)	7406 (59.7)	0.016
No. visits to any clinic in previous 2 years									
0	287 (0.9)	464586 (13.7)	0.56	282 (1.0)	181 (0.7)	0.041	111 (0.8)	49 (0.4)	0.052
1-3	2159 (7.1)	877579 (25.8)	0.53	2084 (7.7)	2286 (8.4)	0.027	902 (6.4)	709 (5.7)	0.028
4-5	1706 (5.6)	401546 (11.8)	0.22	1625 (6.0)	1734 (6.4)	0.017	812 (5.7)	610 (4.9)	0.037
6-10	4731 (15.6)	608226 (17.9)	0.06	4405 (16.2)	4648 (17.1)	0.024	2216 (15.7)	1930 (15.6)	0.003
11-20	7836 (25.8)	537799 (15.8)	0.25	7071 (26.0)	7048 (25.9)	0.002	3630 (25.7)	3144 (25.4)	0.008
≥20	13610 (44.9)	507356 (14.9)	0.67	11764 (43.2)	11334 (41.6)	0.032	6459 (45.7)	5956 (48.0)	0.047
Any hospitalization	4680 (15.4)	274909 (8.1)	0.23	4184 (15.4)	4122 (15.1)	0.006	2283 (16.2)	2103 (17.0)	0.022

Abbreviations: GLP-1RA, glucagon-like peptide-1 receptor agonist; SMD, absolute value of the standardized mean difference; AUDIT-C, Alcohol Use Disorder Identification Test - Consumption; AI/AN, American Indian/Alaska Native; PI/NH, Pacific Islander/Native Hawaiian; COPD, chronic obstructive pulmonary disease; HIV, human immunodeficiency virus; PTSD, post-traumatic stress disorder; VACS, Veterans Aging Cohort Study; HDL, high-density lipoprotein; HbA1c, glycated hemoglobin; eGFR, estimated glomerular filtration rate.

Note: Reported as n (%). Among GLP-1RA recipients in the final analytic cohort, 37% were on liraglutide, 29% on semaglutide, 24% on dulaglutide, 6% on exenatide, and 4% on albiglutide. Median follow-up time was 421 days (interquartile range 222 to 689) for GLP-1RA recipients and 460 days (interquartile range 247 to 728) for unexposed comparators.

Table S2. Characteristics between dipeptidyl-peptidase 4 inhibitor (DPP-4I) recipients and unexposed individuals, before and after propensity score matching and recorded follow-up alcohol consumption.

	Unmatched			Matched			Matched with follow-up AUDIT-C		
	**DPP-4I**	**Unexposed**	**SMD**	**DPP-4I**	**Unexposed**	**SMD**	**DPP-4I**	**Unexposed**	**SMD**
Sample size, n	86190	3397092		77911	77911		44498	40938	
**Demographics**									
Age, years									
20-49	8522 (9.9)	1084052 (31.9)	0.56	7598 (9.8)	6622 (8.5)	0.044	3515 (7.9)	3218 (7.9)	0.001
50-59	16342 (19.0)	510665 (15.0)	0.10	14323 (18.4)	13350 (17.1)	0.033	7560 (17.0)	6937 (16.9)	0.001
60-69	30631 (35.5)	829730 (24.4)	0.24	27833 (35.7)	28329 (36.4)	0.013	16330 (36.7)	15295 (37.4)	0.014
70-74	17816 (20.7)	346176 (10.2)	0.29	16041 (20.6)	16358 (21.0)	0.010	9903 (22.3)	8824 (21.6)	0.017
75-79	6790 (7.9)	234643 (6.9)	0.04	6257 (8.0)	6729 (8.6)	0.022	3727 (8.4)	3558 (8.7)	0.011
≥80	6089 (7.1)	391826 (11.5)	0.15	5859 (7.5)	6523 (8.4)	0.032	3463 (7.8)	3106 (7.6)	0.007
Race/ethnicity									
White	58326 (67.7)	2356397 (69.4)	0.04	52809 (67.8)	52944 (68.0)	0.004	31769 (71.4)	27819 (68.0)	0.075
Black	14606 (16.9)	486723 (14.3)	0.07	13315 (17.1)	13460 (17.3)	0.005	6561 (14.7)	7115 (17.4)	0.072
Hispanic	6507 (7.5)	219207 (6.5)	0.04	5753 (7.4)	5626 (7.2)	0.006	2964 (6.7)	3034 (7.4)	0.029
Asian	1244 (1.4)	33597 (1.0)	0.04	1076 (1.4)	950 (1.2)	0.014	544 (1.2)	506 (1.2)	0.001
AI/AN	547 (0.6)	19995 (0.6)	0.01	483 (0.6)	473 (0.6)	0.002	245 (0.6)	257 (0.6)	0.010
PI/NH	791 (0.9)	22349 (0.7)	0.03	702 (0.9)	629 (0.8)	0.010	369 (0.8)	322 (0.8)	0.005
Mixed race	661 (0.8)	25790 (0.8)	0.00	581 (0.7)	593 (0.8)	0.002	316 (0.7)	308 (0.8)	0.005
Other/unknown	3508 (4.1)	233034 (6.9)	0.12	3192 (4.1)	3236 (4.2)	0.003	1730 (3.9)	1577 (3.9)	0.002
Sex									
Female	3774 (4.4)	250487 (7.4)	0.13	3336 (4.3)	2915 (3.7)	0.028	1734 (3.9)	1578 (3.9)	0.002
Male	82416 (95.6)	3146605 (92.6)	0.13	74575 (95.7)	74996 (96.3)	0.028	42764 (96.1)	39360 (96.1)	0.002
Urban residence	56152 (65.1)	2265441 (66.7)	0.03	50760 (65.2)	50959 (65.4)	0.005	28230 (63.4)	26759 (65.4)	0.040
Census region									
Midwest	21924 (25.4)	758021 (22.3)	0.07	19706 (25.3)	19410 (24.9)	0.009	11791 (26.5)	10190 (24.9)	0.037
Northeast	12978 (15.1)	479431 (14.1)	0.03	11768 (15.1)	11797 (15.1)	0.001	6978 (15.7)	6181 (15.1)	0.016
South	34281 (39.8)	1392728 (41.0)	0.02	31116 (39.9)	31501 (40.4)	0.010	17288 (38.9)	16784 (41.0)	0.044
West	17007 (19.7)	766912 (22.6)	0.07	15321 (19.7)	15203 (19.5)	0.004	8441 (19.0)	7783 (19.0)	0.001
Year of index date									

2009-2012	4071 (4.7)	1009701 (29.7)	0.72	4071 (5.2)	4732 (6.1)	0.037	2205 (5.0)	2420 (5.9)	0.042
2013-2015	11995 (13.9)	780170 (23.0)	0.23	11875 (15.2)	13802 (17.7)	0.067	6881 (15.5)	7289 (17.8)	0.063
2016-2018	31530 (36.6)	831213 (24.5)	0.26	28449 (36.5)	28111 (36.1)	0.009	16322 (36.7)	15015 (36.7)	0.000
2019-2021	38594 (44.8)	776008 (22.8)	0.47	33516 (43.0)	31266 (40.1)	0.059	19090 (42.9)	16214 (39.6)	0.067
**Clinical characteristics**									
Alcohol use disorder	6003 (7.0)	357445 (10.5)	0.13	5668 (7.3)	5668 (7.3)	0.000	3232 (7.3)	2935 (7.2)	0.004
Asthma	2456 (2.8)	67082 (2.0)	0.06	2173 (2.8)	2070 (2.7)	0.008	1278 (2.9)	1207 (2.9)	0.005
Bariatric surgery	65 (0.1)	1422 (0.0)	0.01	56 (0.1)	54 (0.1)	0.001	35 (0.1)	30 (0.1)	0.002
Cancer, localized	7182 (8.3)	240283 (7.1)	0.05	6651 (8.5)	6918 (8.9)	0.012	4055 (9.1)	3800 (9.3)	0.006
Cancer, metastatic	421 (0.5)	20538 (0.6)	0.02	406 (0.5)	406 (0.5)	0.000	257 (0.6)	199 (0.5)	0.013
Cerebrovascular disease	5733 (6.7)	135859 (4.0)	0.12	5353 (6.9)	5655 (7.3)	0.015	3255 (7.3)	3096 (7.6)	0.009
COPD	13381 (15.5)	414178 (12.2)	0.10	12319 (15.8)	12626 (16.2)	0.011	7415 (16.7)	6987 (17.1)	0.011
Congestive heart failure	5739 (6.7)	118003 (3.5)	0.15	5468 (7.0)	6041 (7.8)	0.028	3341 (7.5)	3415 (8.3)	0.031
Dementia	1069 (1.2)	26537 (0.8)	0.05	998 (1.3)	1038 (1.3)	0.005	600 (1.3)	531 (1.3)	0.004
Diabetes, complications	31435 (36.5)	121493 (3.6)	0.92	27837 (35.7)	28070 (36.0)	0.006	16566 (37.2)	15632 (38.2)	0.020
Diabetes, w/o complications	80450 (93.3)	456500 (13.4)	1.87	72212 (92.7)	73566 (94.4)	0.071	41843 (94.0)	39095 (95.5)	0.066
Hemiplegia or paraplegia	387 (0.4)	14848 (0.4)	0.00	367 (0.5)	380 (0.5)	0.002	238 (0.5)	228 (0.6)	0.003
HIV	345 (0.4)	11533 (0.3)	0.01	315 (0.4)	294 (0.4)	0.004	172 (0.4)	175 (0.4)	0.006
Liver disease, mild	5416 (6.3)	112787 (3.3)	0.14	4817 (6.2)	4641 (6.0)	0.009	2870 (6.4)	2643 (6.5)	0.000
Liver disease, moderate to severe	364 (0.4)	12700 (0.4)	0.01	336 (0.4)	382 (0.5)	0.009	221 (0.5)	219 (0.5)	0.005
Myocardial infarction	2285 (2.7)	50430 (1.5)	0.08	2164 (2.8)	2382 (3.1)	0.017	1323 (3.0)	1371 (3.3)	0.021
Opioid use disorder	555 (0.6)	32127 (0.9)	0.03	525 (0.7)	537 (0.7)	0.002	319 (0.7)	300 (0.7)	0.002
Peptic ulcer disease	659 (0.8)	24291 (0.7)	0.01	616 (0.8)	612 (0.8)	0.001	363 (0.8)	340 (0.8)	0.002
Peripheral vascular disease	7557 (8.8)	160397 (4.7)	0.16	7054 (9.1)	7613 (9.8)	0.025	4404 (9.9)	4264 (10.4)	0.017
PTSD	11471 (13.3)	352866 (10.4)	0.09	10198 (13.1)	9750 (12.5)	0.017	6176 (13.9)	5789 (14.1)	0.008
Renal disease	10904 (12.7)	145280 (4.3)	0.31	9902 (12.7)	10477 (13.4)	0.022	6105 (13.7)	5784 (14.1)	0.012
Rheumatic disease	1047 (1.2)	33368 (1.0)	0.02	962 (1.2)	956 (1.2)	0.001	597 (1.3)	538 (1.3)	0.002
Charlson Comorbidity Index									
0	3766 (4.4)	2167254 (63.8)	1.43	3725 (4.8)	2616 (3.4)	0.072	1603 (3.6)	967 (2.4)	0.073
1	30735 (35.7)	564951 (16.6)	0.44	27624 (35.5)	27813 (35.7)	0.005	15249 (34.3)	13970 (34.1)	0.003
2	22901 (26.6)	326179 (9.6)	0.45	20312 (26.1)	20106 (25.8)	0.006	11660 (26.2)	10812 (26.4)	0.005
3	12105 (14.0)	157219 (4.6)	0.33	10967 (14.1)	11290 (14.5)	0.012	6520 (14.7)	6145 (15.0)	0.010

4	8075 (9.4)	79011 (2.3)	0.32	7315 (9.4)	7530 (9.7)	0.009	4447 (10.0)	4124 (10.1)	0.003
≥5	8608 (10.0)	102478 (3.0)	0.29	7968 (10.2)	8556 (11.0)	0.025	5019 (11.3)	4920 (12.0)	0.023
VACS Index									
≤54	7188 (8.3)	665769 (19.6)	0.33	6244 (8.0)	5495 (7.1)	0.036	3000 (6.7)	2703 (6.6)	0.006
>54-61	13916 (16.1)	450956 (13.3)	0.08	12208 (15.7)	11471 (14.7)	0.026	6579 (14.8)	5943 (14.5)	0.008
>61-74	31194 (36.2)	763041 (22.5)	0.30	28104 (36.1)	28256 (36.3)	0.004	16631 (37.4)	15276 (37.3)	0.001
>74	21145 (24.5)	633207 (18.6)	0.14	19728 (25.3)	21383 (27.4)	0.048	11788 (26.5)	11341 (27.7)	0.027
Missing	12747 (14.8)	884119 (26.0)	0.28	11627 (14.9)	11306 (14.5)	0.012	6500 (14.6)	5675 (13.9)	0.021
**Other medications at index date**									
Any alcohol pharmacotherapy	14401 (16.7)	183186 (5.4)	0.37	12883 (16.5)	12819 (16.5)	0.002	7813 (17.6)	7156 (17.5)	0.002
Any neurocognitively-active	36689 (42.6)	864340 (25.4)	0.36	32802 (42.1)	32373 (41.6)	0.011	19583 (44.0)	18219 (44.5)	0.010
Any anticholinergic	14412 (16.7)	313852 (9.2)	0.22	13300 (17.1)	13864 (17.8)	0.019	8045 (18.1)	7684 (18.8)	0.018
Medication count									
0-1	2552 (3.0)	2165621 (63.7)	1.50	2552 (3.3)	2006 (2.6)	0.042	1286 (2.9)	671 (1.6)	0.085
2-4	17245 (20.0)	785028 (23.1)	0.08	17164 (22.0)	18995 (24.4)	0.056	8664 (19.5)	9055 (22.1)	0.065
5-9	48211 (55.9)	395670 (11.6)	0.99	43019 (55.2)	44111 (56.6)	0.028	24785 (55.7)	23768 (58.1)	0.048
≥10	18182 (21.1)	50773 (1.5)	0.71	15176 (19.5)	12799 (16.4)	0.080	9763 (21.9)	7444 (18.2)	0.094
**Substance use**									
Alcohol consumption (AUDIT-C score)									
Low-risk (1-3)	72351 (83.9)	2413092 (71.0)	0.31	65095 (83.6)	65095 (83.6)	0.000	37181 (83.6)	34275 (83.7)	0.005
At-risk (4-7)	11793 (13.7)	787312 (23.2)	0.25	10890 (14.0)	10890 (14.0)	0.000	6281 (14.1)	5719 (14.0)	0.004
Hazardous/binge (≥8)	2046 (2.4)	196688 (5.8)	0.18	1926 (2.5)	1926 (2.5)	0.000	1036 (2.3)	944 (2.3)	0.001
Substance use treatment program visit	5204 (6.0)	320642 (9.4)	0.13	4916 (6.3)	4916 (6.3)	0.000	2815 (6.3)	2562 (6.3)	0.003
Smoking status									
Never	27034 (31.4)	1035672 (30.5)	0.02	24023 (30.8)	23312 (29.9)	0.020	13207 (29.7)	12166 (29.7)	0.001
Former	33891 (39.3)	1090452 (32.1)	0.15	30803 (39.5)	31522 (40.5)	0.019	18448 (41.5)	16813 (41.1)	0.008
Current	25023 (29.0)	1207183 (35.5)	0.14	22847 (29.3)	22837 (29.3)	0.000	12745 (28.6)	11882 (29.0)	0.008
Missing	242 (0.3)	63785 (1.9)	0.17	238 (0.3)	240 (0.3)	0.000	98 (0.2)	77 (0.2)	0.007
**Vital signs**									
Body mass index, kg/m^2									

≤26	9269 (10.8)	942912 (27.8)	0.44	8742 (11.2)	8880 (11.4)	0.006	4813 (10.8)	4504 (11.0)	0.006
>26-32	32235 (37.4)	1411064 (41.5)	0.08	29261 (37.6)	29655 (38.1)	0.010	16667 (37.5)	15570 (38.0)	0.012
>32	41125 (47.7)	855765 (25.2)	0.47	36662 (47.1)	36344 (46.6)	0.008	21263 (47.8)	19420 (47.4)	0.007
Missing	3561 (4.1)	187351 (5.5)	0.06	3246 (4.2)	3032 (3.9)	0.014	1755 (3.9)	1444 (3.5)	0.022
Systolic blood pressure, mm Hg									
≤127	24606 (28.5)	1130585 (33.3)	0.10	22231 (28.5)	22395 (28.7)	0.005	12869 (28.9)	11923 (29.1)	0.005
>127-141	34410 (39.9)	1098716 (32.3)	0.16	30925 (39.7)	30746 (39.5)	0.005	17905 (40.2)	16490 (40.3)	0.001
>141	24617 (28.6)	657807 (19.4)	0.22	22327 (28.7)	23034 (29.6)	0.020	12681 (28.5)	12017 (29.4)	0.019
Missing	2557 (3.0)	509984 (15.0)	0.45	2428 (3.1)	1736 (2.2)	0.055	1043 (2.3)	508 (1.2)	0.084
Diastolic blood pressure, mm Hg									
≤82	60515 (70.2)	1999683 (58.9)	0.24	54876 (70.4)	56095 (72.0)	0.035	32405 (72.8)	30046 (73.4)	0.013
>82	23118 (26.8)	887425 (26.1)	0.02	20607 (26.4)	20080 (25.8)	0.015	11050 (24.8)	10384 (25.4)	0.012
Missing	2557 (3.0)	509984 (15.0)	0.45	2428 (3.1)	1736 (2.2)	0.055	1043 (2.3)	508 (1.2)	0.084
**Laboratory findings**									
Albumin, g/dL									
≤4.0	35077 (40.7)	1043584 (30.7)	0.21	32232 (41.4)	33592 (43.1)	0.035	18445 (41.5)	17847 (43.6)	0.043
>4.0-4.4	30505 (35.4)	1002544 (29.5)	0.13	26991 (34.6)	26117 (33.5)	0.024	15622 (35.1)	13866 (33.9)	0.026
>4.4	12531 (14.5)	549060 (16.2)	0.05	11045 (14.2)	10337 (13.3)	0.026	6290 (14.1)	5300 (12.9)	0.035
Missing	8077 (9.4)	801904 (23.6)	0.39	7643 (9.8)	7865 (10.1)	0.010	4141 (9.3)	3925 (9.6)	0.010
Total cholesterol, mg/dL									
≤137	25437 (29.5)	398730 (11.7)	0.45	22872 (29.4)	23440 (30.1)	0.016	14049 (31.6)	12554 (30.7)	0.020
>137-220	48965 (56.8)	1940768 (57.1)	0.01	44205 (56.7)	44067 (56.6)	0.004	25026 (56.2)	23349 (57.0)	0.016
>220	8069 (9.4)	422070 (12.4)	0.10	7247 (9.3)	6935 (8.9)	0.014	3513 (7.9)	3420 (8.4)	0.017
Missing	3719 (4.3)	635524 (18.7)	0.48	3587 (4.6)	3469 (4.5)	0.007	1910 (4.3)	1615 (3.9)	0.017
HDL cholesterol, mg/dL									
<35	27943 (32.4)	476928 (14.0)	0.44	24726 (31.7)	24119 (31.0)	0.017	14483 (32.5)	12931 (31.6)	0.021
>35-45	31997 (37.1)	860018 (25.3)	0.26	28627 (36.7)	28483 (36.6)	0.004	16405 (36.9)	15010 (36.7)	0.004
>45	22471 (26.1)	1416071 (41.7)	0.33	20915 (26.8)	21779 (28.0)	0.025	11674 (26.2)	11347 (27.7)	0.033
Missing	3779 (4.4)	644075 (19.0)	0.48	3643 (4.7)	3530 (4.5)	0.007	1936 (4.4)	1650 (4.0)	0.016
HbA1c, %									
<6.5	4548 (5.3)	1478790 (43.5)	0.98	4548 (5.8)	4797 (6.2)	0.013	2637 (5.9)	2534 (6.2)	0.011
6.5-<8.0	27149 (31.5)	208018 (6.1)	0.69	26714 (34.3)	31789 (40.8)	0.135	16192 (36.4)	17182 (42.0)	0.114

≥8.0	43939 (51.0)	83437 (2.5)	1.28	36147 (46.4)	30673 (39.4)	0.142	19872 (44.7)	15919 (38.9)	0.117
Missing	10554 (12.2)	1626847 (47.9)	0.81	10502 (13.5)	10652 (13.7)	0.006	5797 (13.0)	5303 (13.0)	0.002
Hemoglobin, g/dL									
≤13.5	24509 (28.4)	643324 (18.9)	0.22	22579 (29.0)	23878 (30.6)	0.036	13276 (29.8)	12787 (31.2)	0.030
>13.5-14.3	16630 (19.3)	496410 (14.6)	0.13	14941 (19.2)	15012 (19.3)	0.002	8743 (19.6)	8082 (19.7)	0.002
>14.3	39548 (45.9)	1653052 (48.7)	0.06	35211 (45.2)	33886 (43.5)	0.034	19668 (44.2)	17575 (42.9)	0.026
Missing	5503 (6.4)	604306 (17.8)	0.36	5180 (6.6)	5135 (6.6)	0.002	2811 (6.3)	2494 (6.1)	0.009
Total bilirubin, mg/dL									
≤0.6	44846 (52.0)	1358736 (40.0)	0.24	40350 (51.8)	40531 (52.0)	0.005	23266 (52.3)	21602 (52.8)	0.010
>0.6	33666 (39.1)	1290881 (38.0)	0.02	30354 (39.0)	30074 (38.6)	0.007	17326 (38.9)	15680 (38.3)	0.013
Missing	7678 (8.9)	747475 (22.0)	0.37	7207 (9.3)	7306 (9.4)	0.004	3906 (8.8)	3656 (8.9)	0.005
Triglycerides, mg/dL									
≤118	24855 (28.8)	1448094 (42.6)	0.29	23164 (29.7)	24407 (31.3)	0.035	13203 (29.7)	12810 (31.3)	0.035
>118-184	24664 (28.6)	724795 (21.3)	0.17	22269 (28.6)	22412 (28.8)	0.004	12871 (28.9)	11878 (29.0)	0.002
>184	32614 (37.8)	561442 (16.5)	0.49	28575 (36.7)	27253 (35.0)	0.035	16337 (36.7)	14447 (35.3)	0.030
Missing	4057 (4.7)	662761 (19.5)	0.48	3903 (5.0)	3839 (4.9)	0.004	2087 (4.7)	1803 (4.4)	0.014
White blood cell count, K/µL									
≤5.7	16572 (19.2)	832569 (24.5)	0.13	15072 (19.3)	15038 (19.3)	0.001	8549 (19.2)	7937 (19.4)	0.004
>5.7-7.2	24910 (28.9)	887653 (26.1)	0.06	22360 (28.7)	22225 (28.5)	0.004	12852 (28.9)	11816 (28.9)	0.000
>7.2	39125 (45.4)	1069377 (31.5)	0.29	35224 (45.2)	35422 (45.5)	0.005	20239 (45.5)	18659 (45.6)	0.002
Missing	5583 (6.5)	607493 (17.9)	0.36	5255 (6.7)	5226 (6.7)	0.001	2858 (6.4)	2526 (6.2)	0.010
Fibrosis-4 score									
<1.45	44492 (51.6)	1529478 (45.0)	0.13	39609 (50.8)	38702 (49.7)	0.023	21887 (49.2)	20343 (49.7)	0.010
1.45-3.25	26424 (30.7)	852004 (25.1)	0.12	24293 (31.2)	25237 (32.4)	0.026	14678 (33.0)	13558 (33.1)	0.003
>3.25	2461 (2.9)	129749 (3.8)	0.05	2328 (3.0)	2588 (3.3)	0.019	1390 (3.1)	1299 (3.2)	0.003
Missing	12813 (14.9)	885861 (26.1)	0.28	11681 (15.0)	11384 (14.6)	0.011	6543 (14.7)	5738 (14.0)	0.020
eGFR, mL/min									
≥60	62645 (72.7)	2444298 (72.0)	0.02	56365 (72.3)	55551 (71.3)	0.023	31783 (71.4)	29245 (71.4)	0.000
30-59	18354 (21.3)	346251 (10.2)	0.31	16601 (21.3)	17238 (22.1)	0.020	9997 (22.5)	9130 (22.3)	0.004
<30	1382 (1.6)	34188 (1.0)	0.05	1338 (1.7)	1605 (2.1)	0.025	795 (1.8)	865 (2.1)	0.024
Missing	3809 (4.4)	572355 (16.8)	0.42	3607 (4.6)	3517 (4.5)	0.006	1923 (4.3)	1698 (4.1)	0.009
**Utilization**									
Index visit in primary care	76271 (88.5)	3247031 (95.6)	0.27	69049 (88.6)	69221 (88.8)	0.007	39241 (88.2)	35786 (87.4)	0.024

No. visits to prescribing clinic in previous 2 years									
0	2362 (2.7)	554547 (16.3)	0.50	2274 (2.9)	1697 (2.2)	0.047	944 (2.1)	484 (1.2)	0.074
1-2	6604 (7.7)	787751 (23.2)	0.44	6177 (7.9)	6568 (8.4)	0.018	2902 (6.5)	2608 (6.4)	0.006
3-4	8956 (10.4)	612358 (18.0)	0.22	8306 (10.7)	8887 (11.4)	0.024	4387 (9.9)	4098 (10.0)	0.005
5-6	10118 (11.7)	419901 (12.4)	0.02	9231 (11.8)	9705 (12.5)	0.019	5127 (11.5)	4806 (11.7)	0.007
7-12	25427 (29.5)	609022 (17.9)	0.27	22783 (29.2)	22565 (29.0)	0.006	13241 (29.8)	12099 (29.6)	0.004
≥12	32723 (38.0)	413513 (12.2)	0.61	29140 (37.4)	28489 (36.6)	0.017	17897 (40.2)	16843 (41.1)	0.019
No. visits to any clinic in previous 2 years									
0	2236 (2.6)	464586 (13.7)	0.43	2149 (2.8)	1535 (2.0)	0.052	881 (2.0)	432 (1.1)	0.077
1-3	8773 (10.2)	877579 (25.8)	0.42	8130 (10.4)	8723 (11.2)	0.025	4006 (9.0)	3573 (8.7)	0.010
4-5	7204 (8.4)	401546 (11.8)	0.12	6595 (8.5)	6871 (8.8)	0.013	3530 (7.9)	3170 (7.7)	0.007
6-10	17854 (20.7)	608226 (17.9)	0.07	16003 (20.5)	16299 (20.9)	0.009	8958 (20.1)	8149 (19.9)	0.006
11-20	23363 (27.1)	537799 (15.8)	0.28	20880 (26.8)	20488 (26.3)	0.011	12247 (27.5)	11200 (27.4)	0.004
≥20	26760 (31.0)	507356 (14.9)	0.39	24154 (31.0)	23995 (30.8)	0.004	14876 (33.4)	14414 (35.2)	0.037
Any hospitalization	9698 (11.3)	274909 (8.1)	0.11	9123 (11.7)	9445 (12.1)	0.013	5473 (12.3)	5509 (13.5)	0.035

Abbreviations: DPP-4I, dipeptidyl-peptidase 4 inhibitor; SMD, absolute value of the standardized mean difference; AUDIT-C, Alcohol Use Disorder Identification Test - Consumption; AI/AN, American Indian/Alaska Native; PI/NH, Pacific Islander/Native Hawaiian; COPD, chronic obstructive pulmonary disease; HIV, human immunodeficiency virus; PTSD, post-traumatic stress disorder; VACS, Veterans Aging Cohort Study; HDL, high-density lipoprotein; HbA1c, glycated hemoglobin; eGFR, estimated glomerular filtration rate.

Note: Reported as n (%). Among DPP-4I recipients in the final analytic cohort, 49% were on saxagliptin, 41% were on alogliptin, 7% were on sitagliptin, and 2% were on linagliptin. Median follow-up time was 547 days (interquartile range 314 to 730) for DPP-4I recipients and 568 days (interquartile range 335 to 730) for unexposed comparators.

Table S3. Characteristics between glucagon-like peptide-1 receptor agonist (GLP-1RA) and dipeptidyl-peptidase 4 inhibitor (DPP-4I) recipients, before and after propensity score matching and recorded follow-up alcohol consumption.

	Unmatched			Matched			Matched with follow-up AUDIT-C		
	**GLP-1RA**	**DPP-4I**	**SMD**	**GLP-1RA**	**DPP-4I**	**SMD**	**GLP-1RA**	**DPP-4I**	**SMD**
Sample size, n	30329	86190		28996	28996		11863	11145	
**Demographics**									
Age, years									
20-49	3990 (13.2)	8522 (9.9)	0.10	3728 (12.9)	3707 (12.8)	0.002	1341 (11.3)	1202 (10.8)	0.017
50-59	6569 (21.7)	16342 (19.0)	0.07	6269 (21.6)	6188 (21.3)	0.007	2505 (21.1)	2286 (20.5)	0.015
60-69	10132 (33.4)	30631 (35.5)	0.04	9703 (33.5)	9652 (33.3)	0.004	4037 (34.0)	3832 (34.4)	0.007
70-74	6633 (21.9)	17816 (20.7)	0.03	6360 (21.9)	6470 (22.3)	0.009	2777 (23.4)	2624 (23.5)	0.003
75-79	2134 (7.0)	6790 (7.9)	0.03	2066 (7.1)	2061 (7.1)	0.001	867 (7.3)	814 (7.3)	0.000
≥80	871 (2.9)	6089 (7.1)	0.20	870 (3.0)	918 (3.2)	0.010	336 (2.8)	387 (3.5)	0.037
Race/ethnicity									
White	21462 (70.8)	58326 (67.7)	0.07	20468 (70.6)	20501 (70.7)	0.002	8398 (70.8)	8045 (72.2)	0.031
Black	4585 (15.1)	14606 (16.9)	0.05	4409 (15.2)	4310 (14.9)	0.010	1777 (15.0)	1592 (14.3)	0.020
Hispanic	2284 (7.5)	6507 (7.5)	0.00	2177 (7.5)	2210 (7.6)	0.004	916 (7.7)	815 (7.3)	0.016
Asian	226 (0.7)	1244 (1.4)	0.07	224 (0.8)	244 (0.8)	0.008	84 (0.7)	87 (0.8)	0.008
AI/AN	198 (0.7)	547 (0.6)	0.00	193 (0.7)	204 (0.7)	0.005	68 (0.6)	70 (0.6)	0.007
PI/NH	265 (0.9)	791 (0.9)	0.00	251 (0.9)	250 (0.9)	0.000	104 (0.9)	77 (0.7)	0.021
Mixed race	227 (0.7)	661 (0.8)	0.00	213 (0.7)	204 (0.7)	0.004	89 (0.8)	76 (0.7)	0.008
Other/unknown	1082 (3.6)	3508 (4.1)	0.03	1061 (3.7)	1073 (3.7)	0.002	427 (3.6)	383 (3.4)	0.009
Sex									
Female	2201 (7.3)	3774 (4.4)	0.12	1990 (6.9)	1912 (6.6)	0.011	801 (6.8)	697 (6.3)	0.020
Male	28128 (92.7)	82416 (95.6)	0.12	27006 (93.1)	27084 (93.4)	0.011	11062 (93.2)	10448 (93.7)	0.020
Urban residence	19332 (63.7)	56152 (65.1)	0.03	18462 (63.7)	18333 (63.2)	0.009	7533 (63.5)	6908 (62.0)	0.031
Census region									
Midwest	7947 (26.2)	21924 (25.4)	0.02	7610 (26.2)	7699 (26.6)	0.007	3032 (25.6)	2999 (26.9)	0.031
Northeast	4134 (13.6)	12978 (15.1)	0.04	3964 (13.7)	3901 (13.5)	0.006	1626 (13.7)	1545 (13.9)	0.005
South	11444 (37.7)	34281 (39.8)	0.04	10985 (37.9)	10905 (37.6)	0.006	4668 (39.3)	4191 (37.6)	0.036
West	6804 (22.4)	17007 (19.7)	0.07	6437 (22.2)	6491 (22.4)	0.004	2537 (21.4)	2410 (21.6)	0.006
Year of index date									

2009-2012	690 (2.3)	4071 (4.7)	0.14	686 (2.4)	713 (2.5)	0.006	279 (2.4)	265 (2.4)	0.002
2013-2015	1569 (5.2)	11995 (13.9)	0.31	1564 (5.4)	1564 (5.4)	0.000	634 (5.3)	655 (5.9)	0.023
2016-2018	10363 (34.2)	31530 (36.6)	0.05	9923 (34.2)	9824 (33.9)	0.007	4215 (35.5)	3804 (34.1)	0.029
2019-2021	17707 (58.4)	38594 (44.8)	0.27	16823 (58.0)	16895 (58.3)	0.005	6735 (56.8)	6421 (57.6)	0.017
**Clinical characteristics**									
Alcohol use disorder	1762 (5.8)	6003 (7.0)	0.05	1685 (5.8)	1685 (5.8)	0.000	728 (6.1)	680 (6.1)	0.001
Asthma	1202 (4.0)	2456 (2.8)	0.06	1127 (3.9)	1116 (3.8)	0.002	501 (4.2)	438 (3.9)	0.015
Bariatric surgery	52 (0.2)	65 (0.1)	0.03	49 (0.2)	35 (0.1)	0.013	22 (0.2)	16 (0.1)	0.010
Cancer, localized	2185 (7.2)	7182 (8.3)	0.04	2105 (7.3)	2101 (7.2)	0.001	933 (7.9)	881 (7.9)	0.001
Cancer, metastatic	106 (0.3)	421 (0.5)	0.02	105 (0.4)	113 (0.4)	0.005	42 (0.4)	51 (0.5)	0.016
Cerebrovascular disease	2350 (7.7)	5733 (6.7)	0.04	2215 (7.6)	2132 (7.4)	0.011	962 (8.1)	922 (8.3)	0.006
COPD	5645 (18.6)	13381 (15.5)	0.08	5323 (18.4)	5277 (18.2)	0.004	2325 (19.6)	2139 (19.2)	0.010
Congestive heart failure	3844 (12.7)	5739 (6.7)	0.21	3438 (11.9)	3159 (10.9)	0.030	1543 (13.0)	1340 (12.0)	0.030
Dementia	381 (1.3)	1069 (1.2)	0.00	357 (1.2)	381 (1.3)	0.007	156 (1.3)	171 (1.5)	0.019
Diabetes, complications	16083 (53.0)	31435 (36.5)	0.33	15112 (52.1)	14865 (51.3)	0.017	6507 (54.9)	6052 (54.3)	0.011
Diabetes, w/o complications	28386 (93.6)	80450 (93.3)	0.01	27262 (94.0)	27441 (94.6)	0.027	11328 (95.5)	10694 (96.0)	0.023
Hemiplegia or paraplegia	160 (0.5)	387 (0.4)	0.01	150 (0.5)	154 (0.5)	0.002	79 (0.7)	76 (0.7)	0.002
HIV	140 (0.5)	345 (0.4)	0.01	130 (0.4)	132 (0.5)	0.001	58 (0.5)	50 (0.4)	0.006
Liver disease, mild	2335 (7.7)	5416 (6.3)	0.06	2206 (7.6)	2126 (7.3)	0.010	964 (8.1)	871 (7.8)	0.011
Liver disease, moderate to severe	126 (0.4)	364 (0.4)	0.00	119 (0.4)	113 (0.4)	0.003	58 (0.5)	59 (0.5)	0.006
Myocardial infarction	1423 (4.7)	2285 (2.7)	0.11	1246 (4.3)	1150 (4.0)	0.017	534 (4.5)	506 (4.5)	0.002
Opioid use disorder	206 (0.7)	555 (0.6)	0.00	194 (0.7)	187 (0.6)	0.003	96 (0.8)	83 (0.7)	0.007
Peptic ulcer disease	223 (0.7)	659 (0.8)	0.00	214 (0.7)	199 (0.7)	0.006	100 (0.8)	72 (0.6)	0.023
Peripheral vascular disease	3490 (11.5)	7557 (8.8)	0.09	3207 (11.1)	3075 (10.6)	0.015	1391 (11.7)	1297 (11.6)	0.003
PTSD	5027 (16.6)	11471 (13.3)	0.09	4735 (16.3)	4646 (16.0)	0.008	2072 (17.5)	1999 (17.9)	0.012
Renal disease	5350 (17.6)	10904 (12.7)	0.14	4956 (17.1)	4717 (16.3)	0.022	2145 (18.1)	1933 (17.3)	0.019
Rheumatic disease	414 (1.4)	1047 (1.2)	0.01	389 (1.3)	391 (1.3)	0.001	163 (1.4)	158 (1.4)	0.004
Charlson Comorbidity Index									
0	1048 (3.5)	3766 (4.4)	0.05	920 (3.2)	786 (2.7)	0.027	249 (2.1)	196 (1.8)	0.025
1	7818 (25.8)	30735 (35.7)	0.21	7681 (26.5)	7969 (27.5)	0.022	2924 (24.6)	2820 (25.3)	0.015
2	8623 (28.4)	22901 (26.6)	0.04	8356 (28.8)	8501 (29.3)	0.011	3456 (29.1)	3280 (29.4)	0.007
3	4566 (15.1)	12105 (14.0)	0.03	4368 (15.1)	4369 (15.1)	0.000	1845 (15.6)	1749 (15.7)	0.004

4	3567 (11.8)	8075 (9.4)	0.08	3366 (11.6)	3318 (11.4)	0.005	1430 (12.1)	1329 (11.9)	0.004
≥5	4707 (15.5)	8608 (10.0)	0.17	4305 (14.8)	4053 (14.0)	0.025	1959 (16.5)	1771 (15.9)	0.017
VACS Index									
≤54	2709 (8.9)	7188 (8.3)	0.02	2608 (9.0)	2614 (9.0)	0.001	958 (8.1)	885 (7.9)	0.005
>54-61	4975 (16.4)	13916 (16.1)	0.01	4808 (16.6)	4789 (16.5)	0.002	1892 (15.9)	1776 (15.9)	0.000
>61-74	10867 (35.8)	31194 (36.2)	0.01	10423 (35.9)	10523 (36.3)	0.007	4364 (36.8)	4179 (37.5)	0.015
>74	7271 (24.0)	21145 (24.5)	0.01	6930 (23.9)	6788 (23.4)	0.012	3033 (25.6)	2766 (24.8)	0.017
Missing	4507 (14.9)	12747 (14.8)	0.00	4227 (14.6)	4282 (14.8)	0.005	1616 (13.6)	1539 (13.8)	0.005
**Other medications at index date**									
Any alcohol pharmacotherapy	7083 (23.4)	14401 (16.7)	0.17	6577 (22.7)	6444 (22.2)	0.011	2826 (23.8)	2699 (24.2)	0.009
Any neurocognitively-active	15455 (51.0)	36689 (42.6)	0.17	14604 (50.4)	14412 (49.7)	0.013	6248 (52.7)	5910 (53.0)	0.007
Any anticholinergic	6647 (21.9)	14412 (16.7)	0.13	6195 (21.4)	5968 (20.6)	0.019	2711 (22.9)	2480 (22.3)	0.014
Medication count									
0-1	912 (3.0)	2552 (3.0)	0.00	832 (2.9)	848 (2.9)	0.003	234 (2.0)	256 (2.3)	0.022
2-4	4800 (15.8)	17245 (20.0)	0.11	4642 (16.0)	4624 (15.9)	0.002	1731 (14.6)	1542 (13.8)	0.022
5-9	15320 (50.5)	48211 (55.9)	0.11	14858 (51.2)	15016 (51.8)	0.011	6008 (50.6)	5653 (50.7)	0.002
≥10	9297 (30.7)	18182 (21.1)	0.22	8664 (29.9)	8508 (29.3)	0.012	3890 (32.8)	3694 (33.1)	0.008
**Substance use**									
Alcohol consumption (AUDIT-C score)									
Low-risk (1-3)	26548 (87.5)	72351 (83.9)	0.10	25301 (87.3)	25301 (87.3)	0.000	10298 (86.8)	9687 (86.9)	0.003
At-risk (4-7)	3296 (10.9)	11793 (13.7)	0.09	3218 (11.1)	3218 (11.1)	0.000	1359 (11.5)	1265 (11.4)	0.003
Hazardous/binge (≥8)	485 (1.6)	2046 (2.4)	0.06	477 (1.6)	477 (1.6)	0.000	206 (1.7)	193 (1.7)	0.000
Substance use treatment program visit	1431 (4.7)	5204 (6.0)	0.06	1375 (4.7)	1386 (4.8)	0.002	599 (5.0)	569 (5.1)	0.003
Smoking status									
Never	9432 (31.1)	27034 (31.4)	0.01	9039 (31.2)	9014 (31.1)	0.002	3671 (30.9)	3389 (30.4)	0.012
Former	12792 (42.2)	33891 (39.3)	0.06	12129 (41.8)	12169 (42.0)	0.003	5005 (42.2)	4793 (43.0)	0.016
Current	8033 (26.5)	25023 (29.0)	0.06	7757 (26.8)	7750 (26.7)	0.001	3173 (26.7)	2949 (26.5)	0.006
Missing	72 (0.2)	242 (0.3)	0.01	71 (0.2)	63 (0.2)	0.006	14 (0.1)	14 (0.1)	0.002
**Vital signs**									
Body mass index, kg/m^2									

≤26	1151 (3.8)	9269 (10.8)	0.28	1148 (4.0)	1179 (4.1)	0.005	446 (3.8)	480 (4.3)	0.028
>26-32	7557 (24.9)	32235 (37.4)	0.27	7489 (25.8)	7594 (26.2)	0.008	3031 (25.6)	2879 (25.8)	0.006
>32	20633 (68.0)	41125 (47.7)	0.41	19382 (66.8)	19211 (66.3)	0.012	8031 (67.7)	7446 (66.8)	0.019
Missing	988 (3.3)	3561 (4.1)	0.05	977 (3.4)	1012 (3.5)	0.007	355 (3.0)	340 (3.1)	0.003
Systolic blood pressure, mm Hg									
≤127	9496 (31.3)	24606 (28.5)	0.06	8956 (30.9)	8983 (31.0)	0.002	3686 (31.1)	3512 (31.5)	0.010
>127-141	12006 (39.6)	34410 (39.9)	0.01	11520 (39.7)	11578 (39.9)	0.004	4721 (39.8)	4454 (40.0)	0.003
>141	8411 (27.7)	24617 (28.6)	0.02	8106 (28.0)	7997 (27.6)	0.008	3372 (28.4)	3078 (27.6)	0.018
Missing	416 (1.4)	2557 (3.0)	0.11	414 (1.4)	438 (1.5)	0.007	84 (0.7)	101 (0.9)	0.022
Diastolic blood pressure, mm Hg									
≤82	22296 (73.5)	60515 (70.2)	0.07	21207 (73.1)	21129 (72.9)	0.006	8891 (74.9)	8351 (74.9)	0.000
>82	7617 (25.1)	23118 (26.8)	0.04	7375 (25.4)	7429 (25.6)	0.004	2888 (24.3)	2693 (24.2)	0.004
Missing	416 (1.4)	2557 (3.0)	0.11	414 (1.4)	438 (1.5)	0.007	84 (0.7)	101 (0.9)	0.022
**Laboratory findings**									
Albumin, g/dL									
≤4.0	14223 (46.9)	35077 (40.7)	0.13	13466 (46.4)	13302 (45.9)	0.011	5625 (47.4)	5260 (47.2)	0.004
>4.0-4.4	9348 (30.8)	30505 (35.4)	0.10	9098 (31.4)	9182 (31.7)	0.006	3690 (31.1)	3546 (31.8)	0.015
>4.4	3592 (11.8)	12531 (14.5)	0.08	3504 (12.1)	3608 (12.4)	0.011	1436 (12.1)	1347 (12.1)	0.001
Missing	3166 (10.4)	8077 (9.4)	0.04	2928 (10.1)	2904 (10.0)	0.003	1112 (9.4)	992 (8.9)	0.016
Total cholesterol, mg/dL									
≤137	10059 (33.2)	25437 (29.5)	0.08	9567 (33.0)	9559 (33.0)	0.001	4097 (34.5)	3900 (35.0)	0.010
>137-220	16132 (53.2)	48965 (56.8)	0.07	15561 (53.7)	15603 (53.8)	0.003	6362 (53.6)	5963 (53.5)	0.003
>220	2454 (8.1)	8069 (9.4)	0.05	2380 (8.2)	2399 (8.3)	0.002	886 (7.5)	808 (7.2)	0.008
Missing	1684 (5.6)	3719 (4.3)	0.06	1488 (5.1)	1435 (4.9)	0.008	518 (4.4)	474 (4.3)	0.006
HDL cholesterol, mg/dL									
<35	11190 (36.9)	27943 (32.4)	0.09	10676 (36.8)	10749 (37.1)	0.005	4380 (36.9)	4207 (37.7)	0.017
>35-45	10763 (35.5)	31997 (37.1)	0.03	10384 (35.8)	10355 (35.7)	0.002	4353 (36.7)	4001 (35.9)	0.017
>45	6661 (22.0)	22471 (26.1)	0.10	6420 (22.1)	6424 (22.2)	0.000	2600 (21.9)	2449 (22.0)	0.001
Missing	1715 (5.7)	3779 (4.4)	0.06	1516 (5.2)	1468 (5.1)	0.007	530 (4.5)	488 (4.4)	0.004
HbA1c, %									
<6.5	2048 (6.8)	4548 (5.3)	0.06	1817 (6.3)	1640 (5.7)	0.026	697 (5.9)	665 (6.0)	0.004
6.5-<8.0	6593 (21.7)	27149 (31.5)	0.22	6482 (22.4)	6641 (22.9)	0.013	2780 (23.4)	2725 (24.5)	0.024

≥8.0	17454 (57.5)	43939 (51.0)	0.13	16668 (57.5)	16678 (57.5)	0.001	6876 (58.0)	6297 (56.5)	0.030
Missing	4234 (14.0)	10554 (12.2)	0.05	4029 (13.9)	4037 (13.9)	0.001	1510 (12.7)	1458 (13.1)	0.011
Hemoglobin, g/dL									
≤13.5	8896 (29.3)	24509 (28.4)	0.02	8457 (29.2)	8400 (29.0)	0.004	3599 (30.3)	3418 (30.7)	0.007
>13.5-14.3	5695 (18.8)	16630 (19.3)	0.01	5473 (18.9)	5476 (18.9)	0.000	2318 (19.5)	2144 (19.2)	0.008
>14.3	13431 (44.3)	39548 (45.9)	0.03	12968 (44.7)	13101 (45.2)	0.009	5180 (43.7)	4907 (44.0)	0.007
Missing	2307 (7.6)	5503 (6.4)	0.05	2098 (7.2)	2019 (7.0)	0.011	766 (6.5)	676 (6.1)	0.016
Total bilirubin, mg/dL									
≤0.6	17664 (58.2)	44846 (52.0)	0.12	16810 (58.0)	16732 (57.7)	0.005	6970 (58.8)	6559 (58.9)	0.002
>0.6	9673 (31.9)	33666 (39.1)	0.15	9424 (32.5)	9560 (33.0)	0.010	3855 (32.5)	3666 (32.9)	0.008
Missing	2992 (9.9)	7678 (8.9)	0.03	2762 (9.5)	2704 (9.3)	0.007	1038 (8.7)	920 (8.3)	0.018
Triglycerides, mg/dL									
≤118	7386 (24.4)	24855 (28.8)	0.10	7131 (24.6)	7114 (24.5)	0.001	2929 (24.7)	2818 (25.3)	0.014
>118-184	8445 (27.8)	24664 (28.6)	0.02	8163 (28.2)	8195 (28.3)	0.002	3391 (28.6)	3184 (28.6)	0.000
>184	12713 (41.9)	32614 (37.8)	0.08	12117 (41.8)	12140 (41.9)	0.002	4979 (42.0)	4632 (41.6)	0.008
Missing	1785 (5.9)	4057 (4.7)	0.05	1585 (5.5)	1547 (5.3)	0.006	564 (4.8)	511 (4.6)	0.008
White blood cell count, K/µL									
≤5.7	4811 (15.9)	16572 (19.2)	0.09	4690 (16.2)	4849 (16.7)	0.015	1887 (15.9)	1875 (16.8)	0.025
>5.7-7.2	8300 (27.4)	24910 (28.9)	0.03	7978 (27.5)	7949 (27.4)	0.002	3320 (28.0)	3067 (27.5)	0.010
>7.2	14873 (49.0)	39125 (45.4)	0.07	14193 (48.9)	14146 (48.8)	0.003	5878 (49.5)	5514 (49.5)	0.001
Missing	2345 (7.7)	5583 (6.5)	0.05	2135 (7.4)	2052 (7.1)	0.011	778 (6.6)	689 (6.2)	0.015
Fibrosis-4 score									
<1.45	17008 (56.1)	44492 (51.6)	0.09	16258 (56.1)	16213 (55.9)	0.003	6577 (55.4)	6185 (55.5)	0.001
1.45-3.25	8080 (26.6)	26424 (30.7)	0.09	7799 (26.9)	7798 (26.9)	0.000	3358 (28.3)	3128 (28.1)	0.005
>3.25	676 (2.2)	2461 (2.9)	0.04	660 (2.3)	666 (2.3)	0.001	305 (2.6)	288 (2.6)	0.001
Missing	4565 (15.1)	12813 (14.9)	0.01	4279 (14.8)	4319 (14.9)	0.004	1623 (13.7)	1544 (13.9)	0.005
eGFR, mL/min									
≥60	20228 (66.7)	62645 (72.7)	0.13	19635 (67.7)	19944 (68.8)	0.023	7941 (66.9)	7625 (68.4)	0.032
30-59	6867 (22.6)	18354 (21.3)	0.03	6533 (22.5)	6426 (22.2)	0.009	2844 (24.0)	2566 (23.0)	0.022
<30	685 (2.3)	1382 (1.6)	0.05	633 (2.2)	589 (2.0)	0.011	271 (2.3)	259 (2.3)	0.003
Missing	2549 (8.4)	3809 (4.4)	0.16	2195 (7.6)	2037 (7.0)	0.021	807 (6.8)	695 (6.2)	0.023
**Utilization**									
Index visit in primary care	22374 (73.8)	76271 (88.5)	0.38	22126 (76.3)	22815 (78.7)	0.057	8942 (75.4)	8537 (76.6)	0.029

No. visits to prescribing clinic in previous 2 years									
0	293 (1.0)	2362 (2.7)	0.14	292 (1.0)	272 (0.9)	0.007	64 (0.5)	67 (0.6)	0.008
1-2	1363 (4.5)	6604 (7.7)	0.13	1350 (4.7)	1434 (4.9)	0.014	341 (2.9)	363 (3.3)	0.022
3-4	1823 (6.0)	8956 (10.4)	0.16	1810 (6.2)	1903 (6.6)	0.013	628 (5.3)	577 (5.2)	0.005
5-6	2144 (7.1)	10118 (11.7)	0.16	2128 (7.3)	2223 (7.7)	0.012	769 (6.5)	761 (6.8)	0.014
7-12	6874 (22.7)	25427 (29.5)	0.16	6784 (23.4)	6951 (24.0)	0.014	2721 (22.9)	2590 (23.2)	0.007
≥12	17832 (58.8)	32723 (38.0)	0.42	16632 (57.4)	16213 (55.9)	0.029	7340 (61.9)	6787 (60.9)	0.020
No. visits to any clinic in previous 2 years									
0	287 (0.9)	2236 (2.6)	0.13	286 (1.0)	280 (1.0)	0.002	61 (0.5)	62 (0.6)	0.006
1-3	2159 (7.1)	8773 (10.2)	0.11	2132 (7.4)	2294 (7.9)	0.021	581 (4.9)	619 (5.6)	0.030
4-5	1706 (5.6)	7204 (8.4)	0.11	1689 (5.8)	1795 (6.2)	0.015	591 (5.0)	580 (5.2)	0.010
6-10	4731 (15.6)	17854 (20.7)	0.13	4663 (16.1)	4768 (16.4)	0.010	1826 (15.4)	1720 (15.4)	0.001
11-20	7836 (25.8)	23363 (27.1)	0.03	7605 (26.2)	7606 (26.2)	0.000	3099 (26.1)	2955 (26.5)	0.009
≥20	13610 (44.9)	26760 (31.0)	0.29	12621 (43.5)	12253 (42.3)	0.026	5705 (48.1)	5209 (46.7)	0.027
Any hospitalization	4680 (15.4)	9698 (11.3)	0.12	4349 (15.0)	4224 (14.6)	0.012	1931 (16.3)	1795 (16.1)	0.005

Abbreviations: GLP-1RA, glucagon-like peptide-1 receptor agonist; DPP-4I, dipeptidyl-peptidase 4 inhibitor; SMD, absolute value of the standardized mean difference; AUDIT-C, Alcohol Use Disorder Identification Test - Consumption; AI/AN, American Indian/Alaska Native; PI/NH, Pacific Islander/Native Hawaiian; COPD, chronic obstructive pulmonary disease; HIV, human immunodeficiency virus; PTSD, post-traumatic stress disorder; VACS, Veterans Aging Cohort Study; HDL, high-density lipoprotein; HbA1c, glycated hemoglobin; eGFR, estimated glomerular filtration rate.

Note: Reported as n (%). Among GLP-1RA recipients in the final analytic cohort, 37% were on liraglutide, 29% on semaglutide, 25% on dulaglutide, 6% on exenatide, and 3% on albiglutide. Among DPP-4I recipients in the final analytic cohort, 55% were on alogliptin, 39% were on saxagliptin, 4% were on sitagliptin, and 2% were on linagliptin. Median follow-up time was 271 days (interquartile range 153 to 457) for GLP-1RA recipients and 285 days (interquartile range 163 to 472) for DPP-4I recipients.

Table S4. Mean (standard error) of pre- and post-index date AUDIT-C scores and difference-in-differences (DiD) across the three treatment groups, stratified by baseline AUD diagnosis.

Baseline AUD diagnosis		GLP-1RAs	Unexposed	DPP-4Is	Unexposed	GLP-1RAs	DPP-4Is
No AUD		n=13249	n=11655	n=41266	n=38003	n=11135	n=10465
	**Pre-index**	1.85 (0.01)	1.90 (0.01)	1.99 (0.01)	2.00 (0.01)	1.84 (0.01)	1.85 (0.02)
	**Post-index**	1.33 (0.01)	1.44 (0.01)	1.51 (0.01)	1.54 (0.01)	1.34 (0.01)	1.42 (0.02)
	**Delta**	-0.52 (0.02)	-0.46 (0.02)	-0.48 (0.01)	-0.46 (0.01)	-0.50 (0.02)	-0.43 (0.02)
	**DiD (95%CI)**	0.06 (0.00, 0.11), p=0.0465		0.02 (-0.01, 0.05), p=0.2701		0.08 (0.02, 0.13), p=0.0101	
AUD		n=881	n=743	n=3232	n=2935	n=728	n=680
	**Pre-index**	4.16 (0.05)	4.27 (0.06)	4.28 (0.03)	4.33 (0.03)	4.13 (0.06)	4.14 (0.06)
	**Post-index**	2.54 (0.05)	3.15 (0.06)	3.08 (0.03)	3.12 (0.03)	2.46 (0.06)	3.13 (0.06)
	**Delta**	-1.62 (0.07)	-1.12 (0.08)	-1.21 (0.04)	-1.22 (0.04)	-1.67 (0.08)	-1.01 (0.08)
	**DiD (95%CI)**	0.51 (0.29, 0.72), p<0.0001		-0.01 (-0.13, 0.11), p=0.8677		0.65 (0.43, 0.88), p<0.0001	

Table S5. Mean (standard error) of pre- and post-index date AUDIT-C scores and difference-in-differences (DiD) across the three treatment groups, stratified by baseline AUDIT-C score.

Baseline AUDIT-C		GLP-1RAs	Unexposed	DPP-4Is	Unexposed	GLP-1RAs	DPP-4Is
1-3 (low risk)		n=12206	n=10698	n=37181	n=34275	n=10298	n=9687
	Pre-index	1.50 (0.01)	1.53 (0.01)	1.54 (0.01)	1.56 (0.01)	1.49 (0.01)	1.50 (0.01)
	Post-index	1.18 (0.01)	1.26 (0.01)	1.29 (0.01)	1.32 (0.01)	1.18 (0.01)	1.26 (0.01)
	Delta	-0.32 (0.02)	-0.27 (0.02)	-0.25 (0.01)	-0.24 (0.01)	-0.32 (0.02)	-0.24 (0.02)
	DiD (95%CI)	0.05 (0.00, 0.09), p=0.0347		0.01 (-0.01, 0.04), p=0.3159		0.08 (0.03, 0.12), p=0.0015	
4-7 (at-risk)		n=1677	n=1480	n=6281	n=5719	n=1359	n=1265
	Pre-index	4.54 (0.03)	4.63 (0.03)	4.56 (0.02)	4.60 (0.02)	4.56 (0.03)	4.58 (0.03)
	Post-index	2.77 (0.03)	3.04 (0.03)	3.08 (0.02)	3.13 (0.02)	2.81 (0.03)	3.07 (0.03)
	Delta	-1.78 (0.04)	-1.59 (0.04)	-1.48 (0.02)	-1.47 (0.02)	-1.75 (0.05)	-1.51 (0.05)
	DiD (95%CI)	0.19 (0.07, 0.31), p=0.0020		0.01 (-0.06, 0.07), p=0.8061		0.24 (0.11, 0.37), p=0.0002	
≥8 (hazardous)		n=247	n=220	n=1036	n=944	n=206	n=193
	Pre-index	9.32 (0.08)	9.41 (0.08)	9.46 (0.04)	9.48 (0.04)	9.38 (0.08)	9.41 (0.09)
	Post-index	3.53 (0.08)	5.00 (0.08)	4.66 (0.04)	4.76 (0.04)	3.60 (0.08)	4.63 (0.09)
	Delta	-5.79 (0.11)	-4.41 (0.11)	-4.81 (0.06)	-4.72 (0.06)	-5.79 (0.12)	-4.78 (0.12)
	DiD (95%CI)	1.38 (1.07, 1.69), p<0.0001		0.09 (-0.07, 0.25), p=0.2790		1.00 (0.68, 1.33), p<0.0001	

Table S6. Mean (standard error) of pre- and post-index date AUDIT-C scores and difference-in-differences (DiD) across the three treatment groups, stratified by baseline BMI.

Baseline BMI (kg/m²)		GLP-1RAs	Unexposed	DPP-4Is	Unexposed	GLP-1RAs	DPP-4Is
<30		n=2956	n=2976	n=17060	n=15718	n=2410	n=2564
	Pre-index	2.12 (0.03)	2.16 (0.03)	2.21 (0.01)	2.25 (0.01)	2.11 (0.03)	2.07 (0.03)
	Post-index	1.48 (0.03)	1.65 (0.03)	1.66 (0.01)	1.71 (0.01)	1.46 (0.03)	1.61 (0.03)
	Delta	-0.64 (0.04)	-0.51 (0.04)	-0.55 (0.02)	-0.54 (0.02)	-0.65 (0.05)	-0.45 (0.04)
	DiD (95%CI)	0.13 (0.01, 0.24), p=0.0335		0.01 (-0.05, 0.06), p=0.7803		0.20 (0.07, 0.33), p=0.0019	
30-39		n=7987	n=7419	n=22660	n=20813	n=6750	n=6722
	Pre-index	2.00 (0.02)	2.05 (0.02)	2.14 (0.01)	2.13 (0.01)	1.98 (0.02)	2.01 (0.02)
	Post-index	1.43 (0.02)	1.55 (0.02)	1.62 (0.01)	1.65 (0.01)	1.43 (0.02)	1.53 (0.02)
	Delta	-0.56 (0.03)	-0.50 (0.03)	-0.52 (0.02)	-0.48 (0.02)	-0.55 (0.03)	-0.47 (0.03)
	DiD (95%CI)	0.06 (-0.01, 0.13), p=0.1121		0.04 (-0.01, 0.08), p=0.1351		0.07 (0.00, 0.15), p=0.0575	
≥40		n=3187	n=2003	n=4778	n=4407	n=2703	n=1859
	Pre-index	1.89 (0.03)	1.83 (0.04)	1.99 (0.03)	2.00 (0.03)	1.88 (0.03)	1.82 (0.04)
	Post-index	1.28 (0.03)	1.34 (0.04)	1.48 (0.03)	1.45 (0.03)	1.30 (0.03)	1.37 (0.04)
	Delta	-0.61 (0.04)	-0.49 (0.05)	-0.51 (0.04)	-0.54 (0.04)	-0.58 (0.04)	-0.45 (0.05)
	DiD (95%CI)	0.12 (-0.01, 0.25), p=0.0687		-0.04 (-0.14, 0.07), p=0.4999		0.13 (0.00, 0.26), p=0.0552	

Table S7. Mean (standard error) of pre- and post-index date AUDIT-C scores and difference-in-differences (DiD) among semaglutide recipients and propensity score-matched unexposed comparators and DPP-4Is, overall and stratified by baseline AUD diagnosis, baseline AUDIT-C score, and baseline BMI.

		Semaglutide	Unexposed	Semaglutide	DPP-4Is
		n=4723	n=4101	n=3744	n=3491
Overall	Pre-index	1.99 (0.02)	2.02 (0.03)	1.97 (0.03)	2.01 (0.03)
	Post-index	1.41 (0.02)	1.56 (0.03)	1.40 (0.03)	1.52 (0.03)
	Delta	-0.58 (0.03)	-0.46 (0.04)	-0.57 (0.04)	-0.49 (0.04)
	DiD (95%CI)	0.12 (0.02, 0.21), p=0.0181		0.08 (-0.02, 0.19), p=0.1108	
Baseline AUD					
No AUD		n=4441	n=3849	n=3526	n=3286
	Pre-index	1.86 (0.02)	1.88 (0.03)	1.84 (0.03)	1.87 (0.03)
	Post-index	1.33 (0.02)	1.45 (0.03)	1.34 (0.03)	1.43 (0.03)
	Delta	-0.53 (0.03)	-0.43 (0.04)	-0.50 (0.04)	-0.44 (0.04)
	DiD (95%CI)	0.10 (0.01, 0.20), p=0.0390		0.06 (-0.04, 0.16), p=0.2420	
AUD		n=282	n=252	n=218	n=205
	Pre-index	4.01 (0.09)	4.15 (0.10)	4.11 (0.10)	4.22 (0.11)
	Post-index	2.69 (0.09)	3.21 (0.10)	2.45 (0.10)	3.03 (0.11)
	Delta	-1.32 (0.13)	-0.94 (0.14)	-1.66 (0.15)	-1.19 (0.15)
	DiD (95%CI)	0.38 (0.00, 0.75), p=0.0484		0.47 (0.05, 0.88), p=0.0270	
Baseline AUDIT-C					
1-3 (low risk)		n=4087	n=3552	n=3259	n=3050
	Pre-index	1.50 (0.02)	1.52 (0.02)	1.50 (0.02)	1.53 (0.02)
	Post-index	1.18 (0.02)	1.28 (0.02)	1.18 (0.02)	1.26 (0.02)
	Delta	-0.32 (0.03)	-0.24 (0.03)	-0.32 (0.03)	-0.27 (0.03)
	DiD (95%CI)	0.08 (0.00, 0.16), p=0.0418		0.05 (-0.03, 0.13), p=0.2199	
4-7 (at-risk)		n=553	n=467	n=418	n=375
	Pre-index	4.46 (0.05)	4.52 (0.06)	4.44 (0.06)	4.56 (0.06)

	Post-index	2.80 (0.05)	3.11 (0.06)	2.79 (0.06)	3.10 (0.06)
	Delta	-1.66 (0.07)	-1.41 (0.08)	-1.65 (0.08)	-1.46 (0.09)
	DiD (95%CI)	0.25 (0.04, 0.46), p=0.0182		0.19 (-0.04, 0.42), p=0.1012	
≥8 (hazardous)		**n=83**	**n=82**	**n=67**	**n=66**
	Pre-index	9.40 (0.13)	9.62 (0.13)	9.45 (0.14)	9.53 (0.14)
	Post-index	3.23 (0.13)	4.88 (0.13)	3.39 (0.14)	4.48 (0.14)
	Delta	-6.17 (0.19)	-4.74 (0.19)	-6.06 (0.20)	-5.05 (0.20)
	DiD (95%CI)	1.42 (0.91, 1.94), p<0.0001		1.01 (0.45, 1.58), p=0.0004	
**Baseline BMI (kg/m^2)**					
<30		**n=1052**	**n=1075**	**n=840**	**n=854**
	Pre-index	2.08 (0.05)	2.13 (0.05)	2.08 (0.05)	2.10 (0.05)
	Post-index	1.48 (0.05)	1.62 (0.05)	1.48 (0.05)	1.62 (0.05)
	Delta	-0.60 (0.07)	-0.50 (0.07)	-0.60 (0.08)	-0.48 (0.08)
	DiD (95%CI)	0.10 (-0.10, 0.29), p=0.3291		0.12 (-0.09, 0.33), p=0.2733	
30-39		**n=2665**	**n=2427**	**n=2105**	**n=2088**
	Pre-index	1.98 (0.03)	2.00 (0.03)	1.96 (0.03)	2.01 (0.03)
	Post-index	1.44 (0.03)	1.58 (0.03)	1.44 (0.03)	1.52 (0.03)
	Delta	-0.54 (0.04)	-0.42 (0.05)	-0.52 (0.05)	-0.49 (0.05)
	DiD (95%CI)	0.12 (-0.01, 0.25), p=0.0626		0.03 (-0.11, 0.16), p=0.7063	
≥40		**n=1006**	**n=599**	**n=799**	**n=549**
	Pre-index	1.90 (0.05)	1.92 (0.07)	1.90 (0.06)	1.85 (0.07)
	Post-index	1.25 (0.05)	1.36 (0.07)	1.22 (0.06)	1.38 (0.07)
	Delta	-0.66 (0.07)	-0.56 (0.09)	-0.68 (0.08)	-0.48 (0.10)
	DiD (95%CI)	0.10 (-0.14, 0.33), p=0.4154		0.20 (-0.04, 0.45), p=0.1039	

Appendices

- **Appendix 1.** Strengthening the reporting of observational studies in epidemiology (STROBE) and reporting of studies conducted using observational routinely collected health data (RECORD) guidelines.

- **Appendix 2.** Alcohol Use Disorders Identification Test – Consumption (AUDIT-C).

Appendix 1. Strengthening the reporting of observational studies in epidemiology (STROBE) and reporting of studies conducted using observational routinely collected health data (RECORD) guidelines.

	**Item No.**	**STROBE items**	**Location in manuscript where items are reported**	**RECORD items**	**Location in manuscript where items are reported**
**Title and abstract**					
	1	(a) Indicate the study's design with a commonly used term in the title or the abstract (b) Provide in the abstract an informative and balanced summary of what was done and what was found	(a) Abstract<br><br>(b) Abstract	RECORD 1.1: The type of data used should be specified in the title or abstract. When possible, the name of the databases used should be included.<br><br>RECORD 1.2: If applicable, the geographic region and timeframe within which the study took place should be reported in the title or abstract.<br><br>RECORD 1.3: If linkage between databases was conducted for the study, this should be clearly stated in the title or abstract.	1.1: Abstract<br><br>1.2: Abstract<br><br>1.3: N/A
**Introduction**					
Background rationale	2	Explain the scientific background and rationale for the investigation being reported	Introduction		
Objectives	3	State specific objectives, including any prespecified hypotheses	Introduction (last paragraph)		
**Methods**					
Study Design	4	Present key elements of study design early in the paper	Methods (section 4.1)		
Setting	5	Describe the setting, locations, and relevant dates, including periods of recruitment, exposure, follow-up, and data collection	Methods (4.1.1, 4.1.2, 4.1.5)		
Participants	6	*(a) Cohort study* - Give the eligibility criteria, and the sources and methods of selection of participants. Describe methods of follow-up<br>*Case-control study* - Give the eligibility criteria, and the sources and methods of case ascertainment and control selection. Give the rationale for the choice of cases and controls	(a) Methods (4.1.1, 4.1.2, 4.1.4, 4.1.5)<br><br>(b) Methods (4.1.4)	RECORD 6.1: The methods of study population selection (such as codes or algorithms used to identify subjects) should be listed in detail. If this is not possible, an explanation should be provided.<br><br>RECORD 6.2: Any validation studies of the codes or algorithms used to select the population should be referenced. If validation	6.1: Methods (4.1.1, 4.1.2)<br><br>6.2: N/A<br><br>6.3: N/A

		Cross-sectional study - Give the eligibility criteria, and the sources and methods of selection of participants<br><br>*(b) Cohort study* - For matched studies, give matching criteria and number of exposed and unexposed<br>*Case-control study* - For matched studies, give matching criteria and the number of controls per case		was conducted for this study and not published elsewhere, detailed methods and results should be provided.<br><br>RECORD 6.3: If the study involved linkage of databases, consider use of a flow diagram or other graphical display to demonstrate the data linkage process, including the number of individuals with linked data at each stage.	
Variables	7	Clearly define all outcomes, exposures, predictors, potential confounders, and effect modifiers. Give diagnostic criteria, if applicable.	Methods (4.1.2, 4.1.3, 4.1.5)	RECORD 7.1: A complete list of codes and algorithms used to classify exposures, outcomes, confounders, and effect modifiers should be provided. If these cannot be reported, an explanation should be provided.	7.1: Methods (4.1.2, 4.1.3, 4.1.5)
Data sources/ measurement	8	For each variable of interest, give sources of data and details of methods of assessment (measurement). Describe comparability of assessment methods if there is more than one group	Methods (4.1.2, 4.1.3, 4.1.5)		
Bias	9	Describe any efforts to address potential sources of bias	Methods (4.1.2, 4.1.4, 4.1.6)		
Study size	10	Explain how the study size was arrived at	Methods (section 4.1), Results (2.1.1)		
Quantitative variables	11	Explain how quantitative variables were handled in the analyses. If applicable, describe which groupings were chosen, and why	Methods (4.1.4, 4.1.6)		
Statistical methods	12	(a) Describe all statistical methods, including those used to control for confounding<br>(b) Describe any methods used to examine subgroups and interactions<br>(c) Explain how missing data were addressed<br>(d) *Cohort study* - If applicable, explain how loss to follow-up was addressed<br>*Case-control study* - If applicable, explain how matching of cases and controls was addressed<br>*Cross-sectional study* - If applicable, describe analytical methods taking account of sampling strategy<br>(e) Describe any sensitivity analyses	(a-c) Methods (4.1.3, 4.1.4, 4.1.6)<br><br>(d) Methods (4.1.5, 4.1.6)<br><br>(e) Methods (4.1.6)		

Data access and cleaning methods		..		RECORD 12.1: Authors should describe the extent to which the investigators had access to the database population used to create the study population.<br><br>RECORD 12.2: Authors should provide information on the data cleaning methods used in the study.	12.1: Methods (4.1.1 Approvals, Data availability)<br><br>12.2: Methods (4.1.3)
Linkage		..		RECORD 12.3: State whether the study included person-level, institutional-level, or other data linkage across two or more databases. The methods of linkage and methods of linkage quality evaluation should be provided.	N/A
**Results**					
Participants	13	(a) Report the numbers of individuals at each stage of the study (*e.g.*, numbers potentially eligible, examined for eligibility, confirmed eligible, included in the study, completing follow-up, and analyzed)<br>(b) Give reasons for non-participation at each stage.<br>(c) Consider use of a flow diagram	(a-c) Results (2.1.1)	RECORD 13.1: Describe in detail the selection of the persons included in the study (*i.e.,* study population selection) including filtering based on data quality, data availability and linkage. The selection of included persons can be described in the text and/or by means of the study flow diagram.	13.1: Results (2.1.1)
Descriptive data	14	(a) Give characteristics of study participants (*e.g.*, demographic, clinical, social) and information on exposures and potential confounders<br>(b) Indicate the number of participants with missing data for each variable of interest<br>(c) *Cohort study* - summarize follow-up time (*e.g.*, average and total amount)	(a) Results (2.1.1), Table S1-S3<br><br>(b) Methods (4.1.4), Table S1-S3<br><br>(c) Methods (4.1.5)		
Outcome data	15	*Cohort study* - Report numbers of outcome events or summary measures over time<br>*Case-control study* - Report numbers in each exposure category, or summary measures of exposure<br>*Cross-sectional study* - Report numbers of outcome events or summary measures	Results (2.1.2)		
Main results	16	(a) Give unadjusted estimates and, if applicable, confounder-adjusted	(a) Results (2.1.2), Figure 2, Methods (4.1.4)		

		estimates and their precision (e.g., 95% confidence interval). Make clear which confounders were adjusted for and why they were included<br>(b) Report category boundaries when continuous variables were categorized<br>(c) If relevant, consider translating estimates of relative risk into absolute risk for a meaningful time period	(b) Methods (4.1.3, 4.1.4, 4.1.5), Table S1-S3<br><br>(c) Table 1-4		
Other analyses	17	Report other analyses done—e.g., analyses of subgroups and interactions, and sensitivity analyses	Results (2.1.2), Figure 2, Figure S2		
**Discussion**					
Key results	18	Summarize key results with reference to study objectives	Discussion (first paragraph)		
Limitations	19	Discuss limitations of the study, taking into account sources of potential bias or imprecision. Discuss both direction and magnitude of any potential bias	Discussion (third and fourth paragraph)	RECORD 19.1: Discuss the implications of using data that were not created or collected to answer the specific research question(s). Include discussion of misclassification bias, unmeasured confounding, missing data, and changing eligibility over time, as they pertain to the study being reported.	19.1 Methods (4.1.4), Discussion (first four paragraphs, final paragraph)
Interpretation	20	Give a cautious overall interpretation of results considering objectives, limitations, multiplicity of analyses, results from similar studies, and other relevant evidence	Discussion (throughout)		
Generalizability	21	Discuss the generalizability (external validity) of the study results	Discussion (second paragraph)		
**Other Information**					
Funding	22	Give the source of funding and the role of the funders for the present study and, if applicable, for the original study on which the present article is based	Funding		
Accessibility of protocol, raw data, and programming code		..		RECORD 22.1: Authors should provide information on how to access any supplemental information such as the study protocol, raw data, or programming code.	Data availability

Appendix 2. Alcohol Use Disorders Identification Test – Consumption (AUDIT-C).

Question	Answer	Score
**1. How often did you have a drink containing alcohol in the past year?**	Never	0 point
	Monthly or less	1 point
	2 to 4 times per month	2 points
	2 to 3 times per week	3 points
	4 or more times per week	4 points
**2. How many drinks containing alcohol did you have on a typical day when you were drinking in the past year?**	0, 1, or 2	0 point
	3 or 4	1 point
	5 or 6	2 points
	7 - 9	3 points
	10 or more	4 points
**3. How often did you have 6 or more drinks on an occasion in the past year?**	Never	0 point
	Less than monthly	1 point
	Monthly	2 points
	Weekly	3 points
	Daily or almost daily	4 points
